# Unearthing the genomes of plant-beneficial *Pseudomonas* model strains WCS358, WCS374 and WCS417

**DOI:** 10.1186/s12864-015-1632-z

**Published:** 2015-07-22

**Authors:** Roeland L. Berendsen, Marcel C. van Verk, Ioannis A. Stringlis, Christos Zamioudis, Jan Tommassen, Corné M. J. Pieterse, Peter A. H. M. Bakker

**Affiliations:** Plant-Microbe Interactions, Department of Biology, Faculty of Science, Utrecht University, Padualaan 8, 3584 CH Utrecht, The Netherlands; Bioinformatics, Department of Biology, Faculty of Science, Utrecht University, Padualaan 8, 3584 CH Utrecht, The Netherlands; Molecular Microbiology, Department of Biology, Faculty of Science, Utrecht University, Padualaan 8, 3584 CH Utrecht, The Netherlands

**Keywords:** Plant growth-promoting rhizobacteria, Induced systemic resistance, Siderophores, Lipopolysaccharides, Antibiotics, Protein secretion system

## Abstract

**Background:**

Plant growth-promoting rhizobacteria (PGPR) can protect plants against pathogenic microbes through a diversity of mechanisms including competition for nutrients, production of antibiotics, and stimulation of the host immune system, a phenomenon called induced systemic resistance (ISR). In the past 30 years, the *Pseudomonas* spp. PGPR strains WCS358, WCS374 and WCS417 of the Willie Commelin Scholten (WCS) collection have been studied in detail in pioneering papers on the molecular basis of PGPR-mediated ISR and mechanisms of biological control of soil-borne pathogens via siderophore-mediated competition for iron.

**Results:**

The genomes of the model WCS PGPR strains were sequenced and analyzed to unearth genetic cues related to biological questions that surfaced during the past 30 years of functional studies on these plant-beneficial microbes. Whole genome comparisons revealed important novel insights into iron acquisition strategies with consequences for both bacterial ecology and plant protection, specifics of bacterial determinants involved in plant-PGPR recognition, and diversity of protein secretion systems involved in microbe-microbe and microbe-plant communication. Furthermore, multi-locus sequence alignment and whole genome comparison revealed the taxonomic position of the WCS model strains within the *Pseudomonas* genus. Despite the enormous diversity of *Pseudomonas* spp. in soils, several plant-associated *Pseudomonas* spp. strains that have been isolated from different hosts at different geographic regions appear to be nearly isogenic to WCS358, WCS374, or WCS417. Interestingly, all these WCS look-a-likes have been selected because of their plant protective or plant growth-promoting properties.

**Conclusions:**

The genome sequences of the model WCS strains revealed that they can be considered representatives of universally-present plant-beneficial *Pseudomonas* spp. With their well-characterized functions in the promotion of plant growth and health, the fully sequenced genomes of the WCS strains provide a genetic framework that allows for detailed analysis of the biological mechanisms of the plant-beneficial traits of these PGPR. Considering the increasing focus on the role of the root microbiome in plant health, functional genomics of the WCS strains will enhance our understanding of the diversity of functions of the root microbiome.

**Electronic supplementary material:**

The online version of this article (doi:10.1186/s12864-015-1632-z) contains supplementary material, which is available to authorized users.

## Background

Plants have evolved elaborate mechanisms to defend themselves against the plethora of pathogens by which they are attacked [1]. Although the interactions between plants and their attackers are often regarded as bilateral, a mesmerizing diversity of microorganisms in and around the plant influences the outcome of the battles between plants and their enemies [2, 3]. Whereas some microbes team up with ‘the bad guys’ [4], many others help to protect the plant and improve plant survival in ecological and agricultural settings [5]. Studies on structure and function of microbiota in and on plant roots have identified a wide array of bacteria and fungi from different taxonomic groups [6–9], many of which possess plant growth-promoting activities [10, 11]. Among the group of plant growth-promoting rhizobacteria (PGPR), the genus *Pseudomonas* is strongly represented. This genus comprises over one hundred species of aerobic bacteria that belong to the γ subclass of the Proteobacteria [12]. Although some *Pseudomonas* spp. are plant pathogenic, many have been found to protect plants by antagonizing soil-borne pathogens through competition for nutrients, production of antimicrobial compounds, or by eliciting a systemic immune response that is effective against a broad spectrum of pathogens, called induced systemic resistance (ISR) [11, 13]. Mutualistic root-colonizing *Pseudomonas* spp. emerged as important players in disease-suppressive soils [7, 14–16], and served as model PGPR in research toward understanding how non-symbiotic root-associated bacteria protect plants against pests and diseases [11, 17].

In the past 30 years, over 300 publications described biological mechanisms involved in the ability of the PGPR strains *Pseudomonas putida* WCS358, *Pseudomonas fluorescens* WCS374, and *P. fluorescens* WCS417 to promote plant growth and health. WCS358, WCS374, WCS417 were isolated in the 1980’s at the Dutch Phytopathological Laboratory “Willie Commelin Scholten” (WCS; [18, 19]) from the rhizosphere of potato (WCS358 and WCS374; [20]) and wheat (WCS417; [21]). They efficiently colonize the rhizosphere of their host plants and have been shown to reduce plant diseases in many plant-pathogen combinations. Originally, the biological control activity of these WCS strains was linked to siderophore mediated competition for iron e.g. [22–28]. Siderophores are small iron-chelating compounds that are secreted by microorganisms under low-iron conditions to enable sequestering and uptake of essential ferric iron from their environment. In most soils, ferric iron is scarcely available as it is present as poorly soluble ferric hydroxides [29]. Microorganisms possess siderophore receptors that specifically recognize and take up their cognate siderophore-iron complexes [30, 31]. In addition to receptors for their own siderophores, many rhizobacteria possess receptors for siderophores produced by other microbes, thereby enhancing their competitiveness under conditions of low iron availability [32, 33].

In addition to their siderophore-mediated antagonistic effect on soil-borne pathogens, the WCS strains also emerged as potent inducers of ISR. Strain WCS417 was among the *Pseudomonas* spp. strains with which the phenomenon of ISR was for the first time experimentally demonstrated [34–36]. With WCS417, ISR was reported for the first time in carnation against Fusarium wilt disease, but since then all three WCS strains have been demonstrated to prime host immunity in different plant species providing enhanced protection against a broad spectrum of plant pathogens and even insect herbivores [13]. Although the three WCS strains are all capable of eliciting ISR, they show host specificity in terms of their ability to induce ISR in different plant species (Table 1). Also within a single plant species, the three WCS strains display differential effectiveness. For instance, in radish*,* WCS374 and WCS358 are potent elicitors of ISR, whereas WCS417 is not [25]. *Arabidopsis thaliana* (Arabidopsis) possesses natural genetic variation for the ability to express WCS417-ISR, a trait that could be mapped to a single genetic locus in the Arabidopsis genome, indicating that rhizobacteria-mediated ISR is genetically determined [37, 38].

**Table 1:** Induction of systemic resistance by Pseudomonas strains WCS358, WCS374, and WCS417

Plant species	WCS358	WCS374	WCS417	Reference
Arabidopsis	**+**	**-** ^**1**^	**+**	[48]
Eucalypt	**+**	**+**	**-**	[131]
Grape vine	**+**	**-**	**+**	[153]
Radish	**-**	**+**	**+**	[25]
Tobacco	**+**	**-**	**-**	[132]
Banana	**?**	**?**	**+**	[154]
Bean	**+**	**?**	**+**	[44, 155]
Carnation	**-**	**?**	**+**	[34, 87]
Rice	**?**	**+**	**?**	[24]
Tomato	**+**	**?**	**+**	[44, 156]

^1^Under specific conditions WCS374 can be manipulated to trigger ISR in Arabidopsis [49, 50]

**?**: not investigated

In the past 20 years, the *Pseudomonas* spp. WCS strains were highly instrumental in research on the molecular basis of rhizobacteria-mediated ISR signaling [13]. Using WCS417, it was demonstrated that the onset of ISR is regulated by the root-specific transcription factor MYB72 [39, 40]. Furthermore, it was shown that establishment of ISR in the leaves functions independently of the plant defense hormone salicylic acid (SA) but instead requires the plant hormones jasmonic acid (JA) and ethylene (ET) [41, 42]. These observations established that WCS417-ISR is mechanistically different from pathogen-induced systemic acquired resistance (SAR; [43]). Different bacterial determinants of the WCS strains have been implicated in the elicitation of ISR, including siderophores, lipopolysaccharides and flagella [44–47]. However, while application of the purified determinants triggers ISR in the host plant, rhizobacterial knockout mutants that no longer produce the respective determinants were not impaired in their ISR-inducing ability [44, 48]. This suggests that bacterial determinants act redundantly in the elicitation of ISR.

The WCS strains can protect different plant species against infection by soil-borne and foliar pathogens, either directly by inhibiting pathogen growth via competition for iron, or indirectly by priming the plant immune system. For both mechanisms, sufficiently high population densities of these rhizosphere bacteria are essential [26]. The density of the WCS strains in the rhizosphere depends on their capacity to efficiently colonize the roots and effectively compete with other microbes in the rhizosphere for nutrients and space. Many of these nutrients are secreted by the plant roots and can manipulate the structure of the microbial community on the root system [2]. In turn, beneficial rhizobacteria can modulate the composition of root excretions and root immune responses, thereby influencing the structure of the root microbiome and the nature of the mutual benefits [40, 49, 50].

Despite the wealth of knowledge on the biology of the *Pseudomonas* spp. WCS strains in relation to their biological control and plant immunity-stimulating activity in host plants, their genomes were not yet elucidated. Here, we report on the whole genome sequences of the *Pseudomonas* strains WCS358, WCS374 and WCS417. We examined these three genomes in the light of the vast amount of functional data that have been published on these strains during the past 30 years. Moreover, we determined the taxonomic position of the WCS strains within the genus *Pseudomonas* and found that each of the three WCS strains is very closely related to one or more other plant-beneficial *Pseudomonas* strains with very different geographic origins. *P. fluorescens* WCS417 appeared to be taxonomically very similar to *Pseudomonas simiae* type strain R81 and was thus renamed into *P. simiae* WCS417. *P. fluorescens* WCS374 and *P. putida* WCS358 belong to the *P. fluorescens* and *P. putida* subgroups, respectively, but are taxonomically not within the 98 % identity range of any previously described type strain. Hence, WCS374 and WCS358 belong to thus far undescribed species, which we propose to name *Pseudomonas defensor* (type strain WCS374) and *Pseudomonas capeferrum* (type strain WCS358), respectively. Analysis of the genomes of WCS358, WCS374 and WCS417 in the light of their previously described biological functions revealed: 1) distinct strategies for siderophore-mediated iron acquisition among the three strains, 2) the structure of the WCS417 siderophore pyoverdine PVD417 and evidence that PVD417 is functionally different from the closely related pyoverdine of WCS374 PVD374; 3) insight into differences and similarities between the bacterial secretion systems and their relevance for rhizosphere competition and interactions with their host; and 4) bacterial determinants that may explain the differential abilities of the three strains to elicit ISR in different plants species. The whole genome sequences of WCS358, WCS374, and WCS417 in combination with the wealth of previously published knowledge on the biology of these PGPR strains will be highly instrumental for the research community working on the mode of action of plant-associated fluorescent *Pseudomonas* spp. in relation to their beneficial effects on plant growth and defense.

## Results

### General genome characteristics

Sequencing of the genomes of WCS358 from the *P. putida* group, and WCS374 and WCS417 from the *P. fluorescens* group was carried out at the Beijing Genome Institute (Beijing, China). A summary of the general sequence characteristics is given in Table 2. The sizes of the genomes varied from approximately 5.94 Mb for WCS358 to 6.09 Mb for WCS374 and 6.17 Mb for WCS417 and they were predicted to contain 5188, 5351 and 5506 coding sequences, respectively. Their G + C contents ranged from 60 to 63.5 %. These characteristics are comparable to those described for taxonomically related and previously sequenced *Pseudomonas* spp. strains [51, 52].

**Table 2:** General sequencing and genome characteristics of the WCS Pseudomonas spp. strains

Characteristics	WCS358	WCS374	WCS417
Genome size (base pairs)	5,940,443	6,085,054	6,169,071
Coverage (fold)	231	271	269
G + C (%)	63.51	60.0	62.7
# Protein coding sequences	5188	5351	5506
Coding (%)	89.1	87.83	88.6
# Contigs	20	11	6
# Scaffolds	8	1	1

### Phylogenetic analysis of WCS358, WCS374, and WCS417

**Fig. 1:**
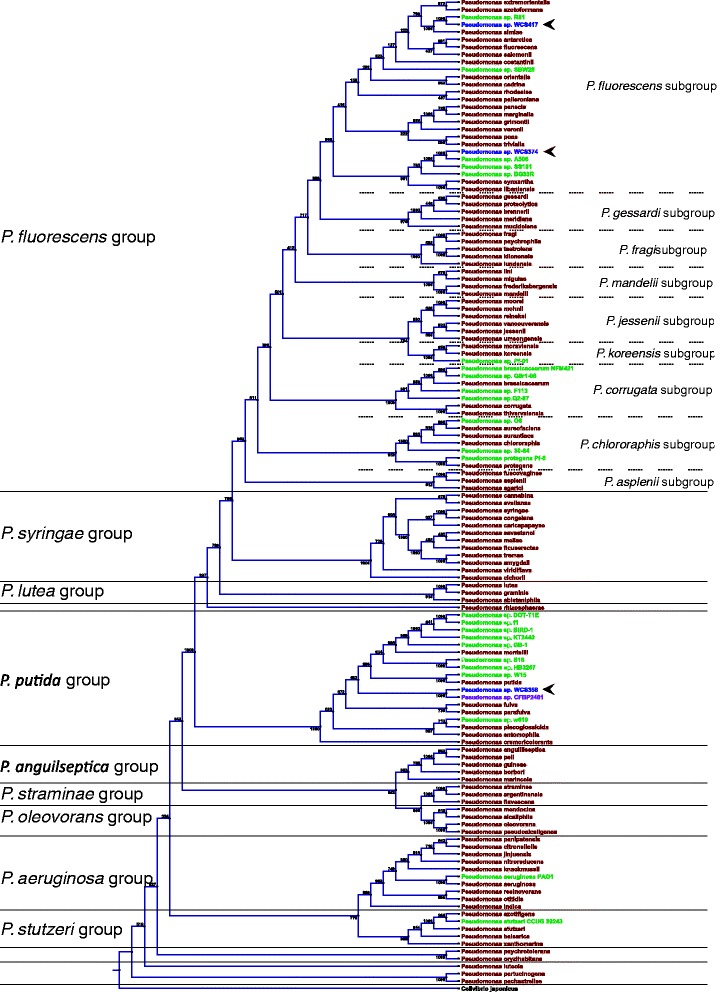
Phylogenetic tree showing the relationship of WCS358, WCS374, and WCS417 with other *Pseudomonas* spp. strains. Phylogenetic tree of the WCS strains (blue; also indicated by arrows) relative to 107 *Pseudomonas* sp. type strains (red), 24 selected *Pseudomonas* sp. strains of which the genomes were already sequenced (green), and *Pseudomonas* sp. strain CFBP2461 (purple). The tree is based on the alignment of concatenated sequences of four core housekeeping genes (*16S rRNA*, *gyrB*, *rpoB*, *rpoD*) of the strains. Bootstrap values from 1000 replicates are indicated at the nodes. Organization of *Pseudomonas* groups and subgroups is according to Mulet *et al.* [53]

The genus *Pseudomonas* is very diverse and is still undergoing taxonomic refinement [53]. Based on their nutritional and physiological characteristics, strains WCS374 and WCS417 were tentatively ascribed to the species *Pseudomonas fluorescens,* whereas WCS358 was described to belong to *Pseudomonas putida* [54, 55]. To obtain a comprehensive overview of the taxonomic position of the WCS strains, the sequences of the core housekeeping genes *16S rRNA*, *gyrB, rpoB* and *rpoD* were compared with the corresponding genes of 107 *Pseudomonas* species type strains [53] and a selection of additional *Pseudomonas* spp. strains of which the full genome was available [51, 52, 56–61]. A phylogenetic tree was generated based on multi-locus sequence analysis (MLSA) of the concatenated sequences of four core housekeeping genes (Fig. 1). This tree shows that both WCS374 and WCS417 are associated with the *P. fluorescens* subgroup as defined by Mulet and co-workers [53]. However, they are not most closely related to the *P. fluorescens* type strain. Similarly, WCS358 is related to the members of the *P. putida* group, but not most closely to the *P. putida* type strain*.* WCS417 has a 100 % nucleotide identity match with the concatenated sequences (NI) of the type strain of *Pseudomonas simiae*. WCS374 is most closely related to the type strain of *Pseudomonas synxantha* (96 % NI), whereas WCS358 shows highest homology with *Pseudomonas monteilii* (94 % NI). As the species boundary for MLSA was proposed to be 97 % [53], WCS417, which was previously ascribed to the *P. fluorescens* species*,* should be regarded as a *P. simiae* strain*,* whereas both WCS374 and WCS358 should be considered representatives of yet undescribed species. WCS417 appears to be closely related to the plant-beneficial *P. simiae* strain R81 (100 % NI), which was also isolated from wheat roots [60]. Furthermore, WCS374 appears to be closely related to two other plant-beneficial *Pseudomonas* spp. strains within its species boundary, i.e. strains A506 (99 % NI; [52]) and SS101 (95 % NI; [52]).

**Fig. 2:**
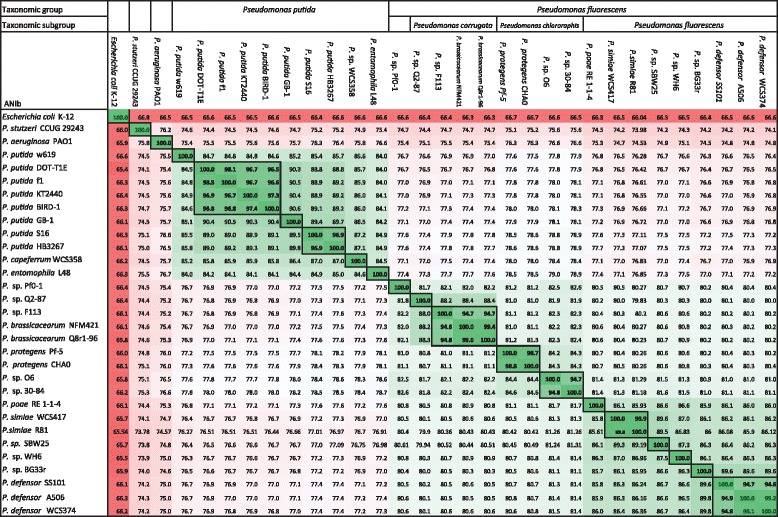
Average Nucleotide Identity based on BLAST for a selection of bacterial genomes. Black lines and bold numbers indicate putative species boundaries. Cell colors indicate similarity scaled from low (red) to high (green). ANIb values were calculated using Jspecies [62]

To further establish the phylogenetic relationship of the WCS strains with other *Pseudomonas* spp. strains, we compared the whole genomes of our strains to available completely sequenced strains of the *P. fluorescens* subgroup and the *P. putida* group by calculating the Average Nucleotide Identity based on BLAST (ANIb) using JSpecies [62]. ANIb values of 94 % [63] and 95 % [62] have been proposed for defining the boundaries between species. The phylogeny based on ANIb values matched with that of MLSA, confirming the phylogenetic separation between strains of the *P. putida* group and the *P. fluorescens* subgroup (Fig. 2). None of the *Pseudomonas* strains for which the genome was available shared more than 94 % of its total genome sequence with WCS358, confirming that WCS358 belongs to a thus far undescribed *Pseudomonas* species within the *P. putida* group. WCS417 shared 99.8 % nucleotide identity with the draft sequence of *Pseudomonas* strain R81, indicating that these strains belong to the same species. Furthermore, *Pseudomonas* spp. strains A506 and SS101 shared 99.1 and 94.8 % of their respective genomes with WCS374 indicating that they belong to the same so far undescribed *Pseudomonas* species within the *P. fluorescens* subgroup (See also Additional file 1: Figure S1 and Additional file 2: Table S1). Together, our genomic analyses indicate that strain WCS417 belongs to the species *P. simiae* of the *P. fluorescens* subgroup, whereas strains WCS358 of the *P. putida* group and WCS374 of the *P. fluorescens* subgroup belong to sofar undescribed species. Here we designate these species as *Pseudomonas capeferrum* (type strain WCS358) and *Pseudomonas defensor* (type strain WCS374; see also Discussion section).

**Fig. 3:**
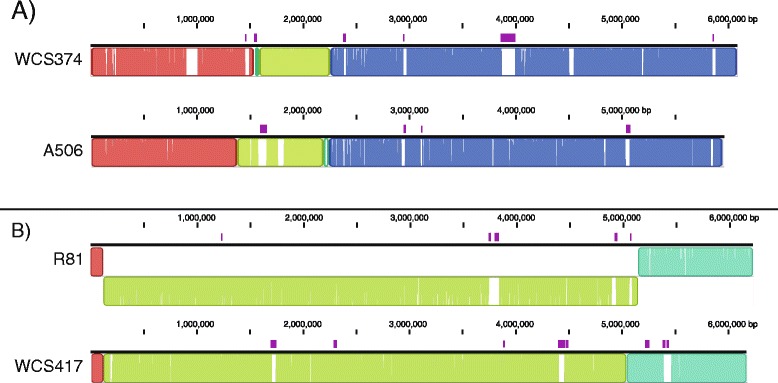
Whole genome alignment of WCS374 and WCS417 with related *Pseudomonas* sp. strains. Whole genome alignments of WCS374 with its close relative *Pseudomonas* sp. strain A506 (**a**) and WCS417 with its close relative *P. simiae* R81 (**b**) were generated with Progressive MAUVE. Colored blocks indicate similar genome regions between the two strains. White gaps indicate genomic regions that are not shared between the compared strains. Genomic islands predicted by Islandviewer are indicated in purple above the black line for each genome. In R81, a large genomic region is represented indented from the rest of the genome as this region was reversely oriented in comparison to WCS417

The whole-genome comparison unearthed that both WCS374 and WCS417 have a close relationship with other *Pseudomonas* spp. strains that were isolated based on their plant-beneficial properties. We aligned the genomes of WCS374 and WCS417 to their closest relative, i.e. *Pseudomonas* spp. strain A506 (isolated from pear phyllosphere, California, USA; [64]) and R81 (isolated from wheat rhizosphere in Bhawanipur, India; [60]), respectively, and found that large parts of the genomes were nearly identical, whereas only small, randomly dispersed genomic regions were missing in the corresponding relatives (Fig. 3). To further analyze the relationship between the pairs of strains, we used Islandviewer [65] to identify genomic islands in the compared genomes. Genomic islands are genomic regions that are derived from horizontal transfers [66]. Figure 3 shows a large overlap between genomic islands and the regions missing in the cognate closest relative, indicating that most of the differences between these closely related strains result from horizontal gene transfer. Although we did not find a close relative of WCS358 among the available sequenced *Pseudomonas* spp. strains, the plant-beneficial *Pseudomonas* sp. strain CFBP2461 (isolated from bean rhizosphere in Angers, France; [67]) was found to produce exactly the same pyoverdine siderophore as WCS358 [68]. Pyoverdines are iron-chelating molecules and their characteristics often closely reflect the phylogeny of the bacteria that produce them. Hence, in the past this feature was often used to characterize bacterial identity, a method known as siderotyping [69]. We amplified the housekeeping genes *16S rRNA*, *gyrB, rpoB* and *rpoD* of CFBP2461 (Genbank accession numbers KM221193- KM221196) and included the concatenated sequence of this strain in an MLSA as described above (Fig. 1). *Pseudomonas* sp. CFBP2461 shared 99.5 % NI with WCS358, indicating that these strains are very similar and should be considered representatives of the same *Pseudomonas* species. Together, these results indicate that for each of the WCS strains other representatives of the same species have been found. The fact that these WCS “look-a-likes” have all been selected in independent searches for plant-beneficial microbes at very different geographic regions suggests that the WCS genome sequences can serve as representative genomes for plant-beneficial *Pseudomonas* spp.

### Siderophores

**Fig. 4:**
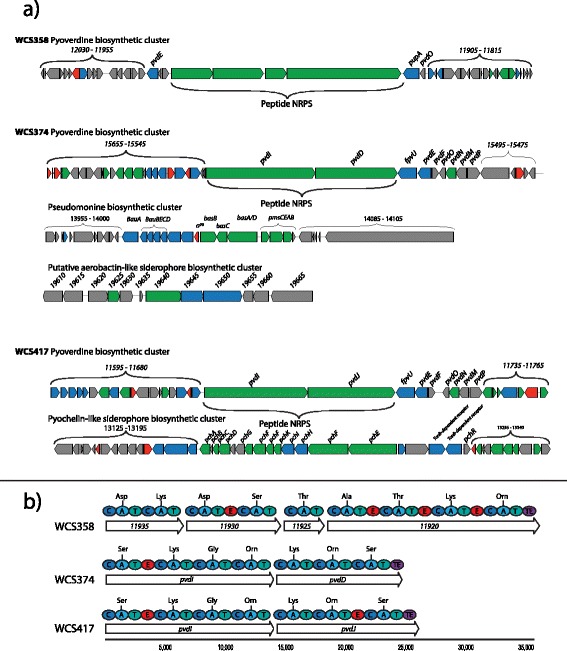
Siderophore biosynthesis genes. (**a**) Siderophore biosynthetic gene clusters in the genomes of WCS358, WCS374, and WCS417 as identified by AntiSMASH. Colors represent different functional gene categories: biosynthetic genes (green); transport-related genes (blue); regulatory genes (red); and other genes (grey). (**b**) Bioinformatic analysis of the NRPSs that synthesize the peptide chain of pyoverdine in the WCS strains. For each NRPS, the domains recognized in the NRPS to function in condensation (C), adenylation (A), thiolation (T), epimerization (E), and epimerization and thioesterase (TE) are shown, as well as the amino acids predicted to be recognized by the adenylation domains

The WCS strains produce siderophores that can inhibit plant pathogens directly via competition for iron, or indirectly via the onset of ISR. Application of purified pyoverdines of WCS358, WCS374 or WCS417 to the plant roots can elicit ISR. However, the pyoverdines of the WCS strains differentially trigger ISR in different plant species [25, 47] suggesting that their structure differs and that these pyoverdines are part of a specific host-microbe recognition system. Whilst the structures of the pyoverdines PVD358 and PVD374 of WCS358 and WCS374, respectively, have been elucidated, the structure of PVD417 of WCS417 is as yet unknown [68, 70]. Pyoverdines consist of a chromophore and a short peptide chain. Both are synthesized by the sequential action of multiple non-ribosomal peptide synthetases (NRPSs). NRPS are multi-modular enzymes in which each module is responsible for addition, attachment or modification of a specific amino acid onto a growing peptide chain [71]. AntiSMASH identified gene clusters that contained NRPSs in all three WCS strains. In each strain, two NRPS-containing gene clusters could be related to the production of pyoverdine. One of these gene clusters is likely to be involved in the synthesis of the chromophore as the only NRPS gene found in this cluster (data not shown) is an ortholog of NRPS *pvdL* of *P. protegens* Pf-5 [72]. The other pyoverdine biosynthesis gene cluster contains NRPS genes that are associated with synthesis of the respective pyoverdine peptide chains (Fig. 4a; indicated in green), pyoverdine transport (Fig. 4a; indicated in blue), or regulation (Fig. 4a; indicated in red). Bioinformatic analysis of the predicted NPRS-mediated peptide chains confirmed the previously elucidated composition of the pyoverdine peptide chains of PVD374 and PVD358 (Fig. 4b). In addition, the bioinformatics analysis predicts that the peptide chain of the WCS417 pyoverdine PVD417 contains the same amino acids as PVD374 in the same order (Ser-Lys-Gly-Orn-Lys-Orn-Ser; Fig. 4b).

Recently, it was demonstrated that WCS374 produces exactly the same PVD as *Pseudomonas* strain *A506* [32, 70]. The peptide chain of the WCS374/A506 PVD was demonstrated to be very similar to that of *Pseudomonas sp.* SBW25 as it contains the same amino acid residues in the same order. It was proposed that the SBW25 PVD differs slightly as its PvdJ contains an epimerization module that is lacking in the orthologous PvdD of the NRPS of WCS374 and A506 and that changed the stereo-isomeric configuration of the 6th amino acid in the chain from L-Orn to D-Orn [32]. Our bioinformatics analysis revealed that the PvdJ ortholog of WCS417 contains a similar epimerization domain that is lacking in WCS374 (Fig. 4b). This suggests that WCS417 produces the same PVD as SBW25, which is a close relative of WCS417 in our phylogenetic analysis (Figs. 1 and 2).

**Fig. 5:**
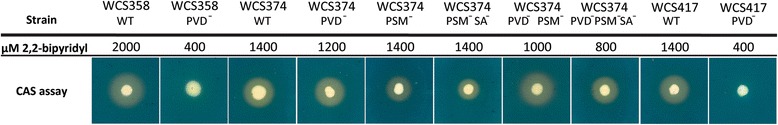
Overview of siderophore production by the WCS strains and their mutant derivatives. Maximum amount of 2,2-bipyridyl in KBA that still allowed growth of strains after 48 h is indicated. Photographs display production of an orange halo by the bacterial strains on CAS agar, which is indicative for siderophore production. Abbreviations stand for Wild type (WT), pyoverdine (PVD), pseudomonine (PSM), pyochelin (PCH) or salicylic acid (SA). Production of pyochelin by WCS417 has not been demonstrated

Pyoverdine knockout mutants WCS358-PVD^−^ and WCS417-PVD^−^ are completely abolished in siderophore activity in the universal siderophore assay on CAS agar medium (Fig. 5) [73–75], indicating that both strains produce only one type of siderophore. However, pyoverdine knockout mutant WCS374-PVD^−^ still produced a halo on CAS medium (Fig. 5) [75], indicating that this strain produces one or more additional siderophores. Mercado-Blanco *et al.* [76], identified a second siderophore in WCS374, called pseudomonine (PSM). Pseudomonine is composed of salicylic acid (SA), cyclothreonine and histamine. Although microbially produced SA has iron-chelating properties and is implicated to function as a siderophore itself [77], the latter was questioned by other studies [78, 79]. Mercado-Blanco and co-workers [76] identified the *pmsCEAB* operon in WCS374 as responsible for production of SA and pseudomonine. Matthijs and co-workers [80] identified two NRPS genes upstream of *pmsCEAB*, i.e. *basB* and *basAD*, which are thought to function in the adenylation and cyclization of the amino acid threonine and subsequent assembly of pseudomonine [81]. All the genes required for pseudomonine biosynthesis were also detected in the genome sequence of WCS374 (Fig. 4a). Interestingly, mutants of WCS374 that do not produce PVD374, PSM or SA, still produce a halo in the CAS assay and are able to grow on KBA with 800 μM of the iron chelator bipyridyl, whereas the pyoverdine-deficient mutants WCS417-PVD^−^ and WCS358-PVD^−^ can only tolerate 400 μM of 2,2-bipyridyl (Fig. 5). This indicates that WCS374 produces yet another siderophore. Searching the WCS374 genome for additional siderophore biosynthesis genes using antiSMASH revealed a gene cluster (PD374_19610 - PD374_19665) involved in the production and transport of an aerobactin-like siderophore (Fig. 4a). This gene cluster is also present in the genomes of the WCS374 relatives SS101 and A506, although the ortholog of PD374_19640, which is related to *IucC/IucA* and is putatively required for the production of the siderophore, contains a frameshift mutation in A506.

Leeman *et al.* [25] investigated the production of SA by the three WCS strains and found that besides WCS374 also WCS417 is capable of producing SA. To identify the corresponding biosynthesis gene cluster in WCS417, the WCS374 genes *pmsC* and *pmsB* were used as bait in a BLASTp search of the WCS417 genome. This revealed two WCS417 orthologs (PS417_13200 and PS417_13205) in a genomic region that was identified by antiSMASH as being putatively responsible for the production of a pyochelin-like siderophore (Fig. 4a). Like pseudomonine, pyochelin is an iron-chelating siderophore found in *Pseudomonas* spp. that comprises a SA moiety [82]. However, pyoverdine knockout mutant WCS417-PVD^−^ does not show any siderophore activity on CAS agar at 28 °C, nor can it tolerate higher levels of bipyridyl than the pyoverdine-deficient mutant WCS358-PVD^−^. Thus, it is unlikely that a pyochelin-like siderophore is indeed produced by WCS417 under the growth conditions tested.

Microbially produced siderophores are re-absorbed after they have complexed ferric iron from the environment. This is mediated by TonB-dependent proteins (TBDPs) that specifically recognize and transport siderophore-iron complexes into the periplasm [30]. We identified 40 TBDP-encoding genes in WCS358, 31 in WCS374 and 33 in WCS417. A minority of these 104 TBDPs also contain a short N-terminal domain, which is typical for TBDP-mediated transduction of environmental signals to the cytoplasm [83]. Most bacteria contain less than 14 TBDPs in their proteome, although some bacteria have larger numbers [32]. Recently, Hartney and co-workers [32] demonstrated that *P. protegens* Pf-5 contains 45 TBDPs. Six of these TBDPs (FpvU to FpvZ) function as ferric-pyoverdine receptors (FPVs) that facilitate the uptake of specific pyoverdine-iron complexes and enable this bacterium to use not only its native pyoverdine, but also heterologous pyoverdines from other strains. Previously it was reported that TBDPs cluster according to their substrate rather than to phylogeny [72]. Because, the ferric-pyoverdine receptors form a clear clade within the TBDPs [32, 84], we used the corresponding *Fpv* genes of strain Pf-5 to identify pyoverdine receptor genes in the three WCS strains. The amino acid sequences of the identified putative pyoverdine receptors were aligned together with the amino acid sequences of the six previously described pyoverdine receptors of Pf-5, after which a phylogenetic tree was build (Additional file 3: Figure S2). The six Pf-5 FPV pyoverdine receptors clustered together, confirming previous findings [72] and this cluster included 10 TBDPs of WCS358, five of WCS417 and four of WCS374.

Using combinations of deletion mutants of the six *Fpv* genes of Pf-5 it is possible to identify which FPV pyoverdine receptors are required for the uptake of specific ferric-pyoverdines [32]. Previously, it was demonstrated that Pf-5 can utilize ferric-pyoverdine of WCS374 and A506 through the receptors FpvU and FpvY, while ferric-pyoverdine of SBW25 can only be utilized through receptor FpvU. This discrepancy was attributed to the different stereo-isomeric configuration of the ornithine residue on position 6 of the pyoverdine peptide chain (Fig. 4b). Because from the genome sequence of WCS417 we predicted that PVD417 would be similar to pyoverdine of SBW25, we set out to functionally validate this using the set of Pf-5 *fpv* mutants [32]. To this end, the six *fpv* deletion mutants of Pf-5 were tested under low-iron conditions for their capacity to be cross-fed by WCS358, WCS374, or WCS417. In line with the descriptions of the specific nature of PVD358 [33, 68], none of the *fpv* mutants of Pf-5 could use the pyoverdine produced by WCS358 (Additional file 4: Figure S3). However, all mutants were able to grow in the presence of WCS374, confirming previous findings by Hartney and co-workers [32]. In the presence of WCS417, all *fpv* mutants of Pf-5 were able to grow, except mutant *fpvU*, indicating that pyoverdin receptor FpvU is required for the uptake of ferric-PVD417, which resembles the findings for SBW25. These results confirm the prediction from our bioinformatical analysis that WCS417 produces the same pyoverdin as SBW25 and highlights that slight differences in pyoverdine structure can have implications for specificity in heterologous uptake of iron-pyoverdin complexes.

### Lipopolysaccharides

Lipopolysaccharides (LPS) are molecules in the outermembrane of Gram negative bacteria, and are recognized as microbe-associated molecular patterns (MAMPs) that are able to elicit immune responses in plants and animals [85, 86]. Application of purified LPS of WCS417 to the roots of carnation plants was shown to trigger ISR, resulting in reduced Fusarium wilt disease when Fusarium was inoculated in the stem [87]. In radish, both WCS374 and WCS417 can trigger ISR, whereas WCS358 cannot. This differential effectiveness of ISR inducibility could be attributed to strain-specific differences in LPS, because in radish purified LPS of WCS417 and WCS374 triggered ISR, whereas that of WCS358 did not [88]. Bacterial LPS usually consists of three domains [86]. The first domain, lipid A, consists of a bisphosphorylated glucosamine-disaccharide backbone substituted with several fatty acids, which anchors the LPS to the outer membrane. Attached to lipid A is the LPS core, an oligosaccharide of about 9–10 sugars, which may be extended with the O-antigen or O-chain. The O-chain is a polysaccharide comprised of repeating units. The number and nature of sugars in the O-chain units is highly specific and can differ dramatically even between strains of the same species [86]. Under conditions of high iron availability, mutants of WCS374 and WCS417 that lack the O-antigen of the LPS (WCS374-ΔOA and WCS417-ΔOA) were no longer able to elicit ISR in radish, demonstrating that under these conditions the LPS of these strains is the only bacterial determinant implicated in ISR and that the O-antigen is the active component [88]. The importance of the O-chain of the LPS was also demonstrated in Arabidopsis. In this plant species, strains WCS417 and WCS358 are able to elicit ISR against *Pseudomonas syringae*, whereas strains WCS374 cannot [48]. Using LPS-containing cell envelopes of these strains, and mutants lacking the O-antigen it was shown that the O-chains of WCS417 and WCS358 are ISR-eliciting bacterial determinants, and that the O-antigen of the LPS of the three WCS strains are differentially recognized in Arabidopsis and radish.

**Fig. 6:**
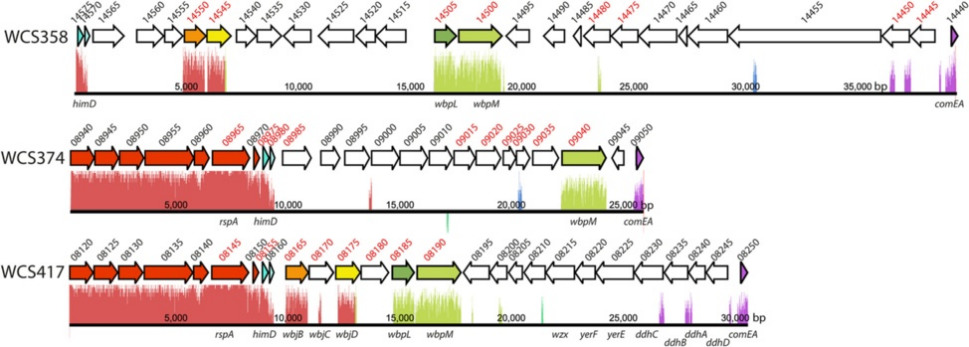
LPS O-antigen biosynthetic loci. Putative LPS O-antigen biosynthetic loci (OBL) in the genomes of WCS358, WCS374, and WCS417 as identified in a BLASTp search using 20 OBL identified in *P. aeruginosa* as bait. Genes of which the gene tags are designated in red font are orthologs of genes found in at least one of the 20 *P. aeruginosa* OBL. Colors of the arrows indicate shared orthologs in the three WCS strains as determined with reciprocal BLASTp. Graphics under the arrows represent a measure of similarity as determined with Progressive MAUVE

In order to identify genes of the WCS strains involved in the biosynthesis of the highly variable O-antigen of LPS, we searched for putative O-antigen biosynthetic loci (OBL) using different OBL described for *P. aeruginosa* as bait. In *P. aeruginosa*, 20 distinct O-antigen serotypes are known for which the biosynthetic loci have been sequenced [89]. The WCS genomes were mined for orthologs of the predicted proteins in these 20 *P. aeruginosa* OBL. In each of the WCS strains, only one gene cluster was found with genes coding for more than four orthologs of the *P. aeruginosa* OBL proteins (Fig. 6). In *P. aeruginosa*, an OBL encoding the major enzymes for O-antigen biogenesis is usually found in between the highly conserved genes *himD* and *wbpM*. Orthologs for these two genes were also found in all the OBL of the WCS strains, although in the WCS strains genes encoding orthologs of the OBL proteins were also found upstream of *himD* (Fig. 6). The region upstream of *himD* is highly conserved between WCS417 and WCS374, but contains an ortholog of rspA that encodes an essential component of the protein synthesis machinery of *Escherichia coli* [90] and is most likely not involved in LPS biosynthesis.

Previously, the composition of the O-antigens of WCS374 and WCS358 were investigated biochemically and reported to consist of glucose and fucose (WCS374), and glucose and quinovosamine (WCS358), respectively [91]. However, the predicted function of the genes in the OBL of WCS374 and WCS358 are not clearly related to the biosynthesis of such subunits. Furthermore, the complexity of especially the OBL of WCS358 suggests that the O-antigen composition is not as clear-cut as described by De Weger and co-workers [91]. In contrast to the O-antigens of WCS358 and WCS374, the composition of the WCS417 O-antigen has not been previously explored. The genome sequence of the WCS417 OBL gives a number of clues towards the structure of the WCS417 LPS O-antigen. The WCS417 genes with locus tags PS417_08220 – PS417_08245 are homologs of *ddhA-ddhD*, *yerE, and yerF*, which are required for the synthesis of the O-antigen subunit yersiniose in *Yersinia pseudotuberculosis* [92, 93] a sugar that has been detected before in a *Pseudomonas* spp. O-antigen [94]. The presence of the genes with locus tags PS417_08165 – PS417_08175, which encode orthologs of WbjB, WbjC and WbjD that are required for the synthesis of the O-chain component N-acetyl-L-fucosamine (L-FucNAc) [95], suggests that L-FucNAc is also part of the WCS417 O-antigen. In addition, an ortholog of *wbpM* of *P. aeruginosa* is also found in the WCS417 OBL. WbpM catalyzes a reaction that ultimately results in either 2-acetamido-2-deoxy-D-fucose (D-FucNAc) or 2-acetamido-2-deoxy-D-quinovose (D- QuiNAc) [86]. It is therefore likely that the WCS417 LPS O-antigen contains yersiniose, L-FucNAc and D-QuiNAc or D-FucNAc. Within the WCS417 OBL, only four genes are shared between WCS417 and WCS358 (Locus tags PS417_08165, PS417_08175, PS417_08185 and PS417_08190) and only one of them (PS417_08190) is also present in WCS374 (Fig. 6). This lack of homology between the OBLs of the three WCS strains is likely to result in variation in O-chain composition, which may be causally related to the observed host-microbe specificity of LPS in eliciting ISR.

### Antimicrobial compounds

Meziane and co-workers [44] investigated the redundancy of ISR-eliciting bacterial determinants in different plant species. They found that WCS358 could elicit ISR against *Botrytis cinerea* in tomato*.* This ISR could also be triggered by applying purified LPS or pyoverdine to the roots but not by applying flagella of WCS358. Nonetheless, a double knockout derivative of WCS358 that did not produce the LPS O-antigen or PVD358 still elicited ISR*.* This indicated that at least a third WCS358 determinant is able to elicit ISR in tomato [44]. In addition to siderophores, LPS and flagella, antimicrobial compounds are bacterial determinants that can induce systemic resistance [45, 96–98]. However, to date no antimicrobial metabolites have been described for the three WCS strains under investigation. Tran and co-workers [96] demonstrated that the cyclic lipopeptide massetolide A, produced by *Pseudomonas* strain SS101, can induce ISR. Like siderophores, cyclic lipopeptides are non-ribosomally produced by NRPSs. All NRPS-containing gene clusters identified by antiSMASH in the genomes of the three WCS strains could be related to the production of the siderophores described above, except for one gene cluster in WCS358 (PC358_04000 - PC358_4180). This gene cluster putatively encodes three NRPS that share 100 % identity in a BLASTp search against PsoA, PsoB and PsoC. PsoA-C are responsible for the biosynthesis of the cyclo lipopeptides putisolvin I and II in *P. putida* PCL1445 [99]. In PCL1445, three additional genes have been shown to be important for putisolvin production. The LuxR-type regulator gene *psoR* is located upstream of *psoA* and is required for expression of the *pso* cluster, whereas *macA* and *macB*, located downstream of *psoC*, are involved in putisolvin production or export [99, 100]. Interestingly, also the entire *psoR-macB* cluster of PCL1445 shares 100 % nucleotide identity with WCS358. Tot test if WCS358 is indeed capable of producing these cyclic lipopeptides, a drop collapse assay was performed. In this assay, WCS358 was able to reduce surface tension, whereas WCS374 and WCS417 were not, demonstrating that WCS358 indeed produces a surfactant (Additional file 5: Figure S4).

Besides the putisolvin biosynthesis cluster, other genes that are potentially involved in the synthesis of compounds with broad-spectrum antibiotic activity could not be identified in the genomes of WCS358, WCS374 and WCS417. For instance, biosynthesis genes for the antimicrobial compounds 2,4-diacetylphloroglucinol, phenazines, hydrogen cyanide, and pyrrolnitrin, which are abundantly present in root-associated *Pseudomonas* strains [52, 82], are not present in the genomes of the sequenced WCS strains. This corroborates with early observations that the *in vitro* antagonistic activity of these strains was only apparent at low-iron conditions, which suggested that the observed antagonistic activity of the WCS strains is predominantly based on siderophore-mediated competition for iron [20, 21]. However, some genes were detected that encode putative bacteriocins. Bacteriocins are bacteriocidal proteins that are generally effective against a narrow taxonomic range of bacteria closely related to the producer [101]. We mined the WCS genomes with BAGEL3 [102] for orthologs of bacteriocin-encoding genes that were previously identified in taxonomically related strains [52]. Four putative bacteriocins were identified in WCS417, one related to S-type pyocins of *P. aeruginosa* (PS417_07930, pyocin AP41-like) and three to R-type pyocins (PS417_05796, PS417_10225 and PS417_05705). In WCS374, three bacteriocins were identified: again one related to S-type pyocins (PD374_22800, pyocin S6-like) and two to R-type pyocins (PD374_05705 and PD374_06430). In WCS358, no bacteriocins could be identified. Together, the genomes of WCS358, WCS374, and WCS417 indicate that these *Pseudomonas* strains produce a relatively small pallet of known antibiotics, confirming that their plant protective capacity is rather based on other mechanisms (e.g. siderophore-mediated competition for iron and ISR).

### Protein secretion systems

In order to deal with different environments, competing microbes and accommodating hosts, bacteria need to secrete enzymes and other proteins into the extracellular environment [103]. Five different protein secretion systems (type I, II, III, V, and VI) are typically found within the *Pseudomonas* genus [104]. Differences in these protein secretion systems are likely to be important for the excretion of proteins involved in traits that influence rhizosphere competence of the WCS strains and for delivery of putative ISR-eliciting determinants.

Type I secretion systems consist of three components that together span the bacterial cell envelop and transport their products from the cytosol directly to the extracellular environment [104]. To identify gene clusters in the WCS genomes that encode the Type I secretion system (T1SS), a BLASTP search was performed with protein sequences encoded by the *P. aeruginosa aprDEF* gene cluster as bait. Two complete T1SS loci were found in the WCS417 genome (Additional file 6: Table S2). One T1SS gene cluster contained genes encoding a lipase and an ortholog of the alkaline protease AprA [105], whereas the second T1SS gene cluster contained a gene encoding an ortholog of the hemophore HasA [106]. It is likely that the T1SSs are dedicated to the secretion of these proteins that are encoded in their gene cluster. Also in WCS374, the genes for two complete T1SSs were found of which one also shared an operon with *aprA* and a lipase. For the second T1SS in WCS374 and for both T1SSs found in WCS358, the substrate was not so obvious, as the corresponding gene clusters did not provide strong clues (Additional file 6: Table S2). For instance, no ortholog of AprA could be found in the WCS358 genome. To experimentally confirm this, we used a milk powder assay to detect AprA protease activity. WCS374 and WCS417, which possess an *aprA* gene in one of their T1SS gene clusters, both showed a clearing zone around their colonies when grown on KBA supplemented with milk powder. For *P. syringae* pv. *tomato* DC3000, such a clearing zone could be attributed to AprA activity as its *aprA* mutant did not show a halo (Additional file 7: Figure S5). Also WCS358 lacked such extracellular protease activity, confirming previous observations [107].

Unlike the T1SS, proteins secreted by type II secretion systems (T2SS) do not directly transfer the secreted proteins over the entire envelop, but make a stopover in the periplasmic space [104]. To identify gene clusters that encode the Type II secretion system (T2SS) that transports proteins across the outermembrane, a BLASTp search was performed using protein sequences encoded by the *P. aeruginosa xcpP-xcpZ* and *hxcP-hxcZ* gene clusters. Clusters containing four or more genes with significant similarity to the bait genes (e-value <10^−5^) were considered T2SS loci. In this way, two T2SS loci were found in the WCS417 genome, whereas the WCS374 genome seemed to contain only one T2SS locus (Additional file 6: Table S2). Also in WCS358, only a single T2SS locus was found using the *P. aeruginosa* T2SSs as baits. This WCS358 T2SS locus resembled the Xcp T2SS of *P. aeruginosa* PA01, which was recently found to secrete the PhoX-type phosphatase UxpB under phosphate-limited conditions, thereby stimulating the phosphate uptake machinery of the bacterium [108]. A second T2SS of WCS358 was detected using the Xcm cluster described in *P. putida* GB-1 and KT2440 as bait (88 % nucleotide identity shared with the cluster of GB-1; [109]). Activity of this T2SS has not been confirmed in WCS358.

There is a strong resemblance between T2SS and the machinery required for type IV pili biogenesis [110]. In WCS358, type IV pilus genes were previously investigated, because type IV pili might have a role in attachment of bacteria to the root surface [111]. Even though type IV pili could not be detected on the surface of WCS358, a cluster containing orthologs of the *pilA*, *pilC* and *pilD* genes of *P. aeruginosa* PAO1 could be identified. However, a gene for an ortholog of the traffic ATPase PilB was lacking in this gene cluster. In the complete genome of WCS358, we could detect five genes encoding proteins with significant homology to *P. aeruginosa* PAO1 PilB. However, the PilB ortholog with the highest similarity (PC358-2543; 43 % AA identity) was found to be XcpR encoded in the WCS358 Type II secretion *xcp* gene cluster. A PilB ortholog is apparently not present in WCS358, which explains the lack of type IV pili in WCS358.

The type III secretion system (T3SS), which consists more than 20 proteins, is the most complex of all known protein secretion systems found in bacteria [104]. In the genomes of WCS417 and WCS374, ORFs encoding structural and regulatory components of the T3SS are organized in 26-kb and 18-kb clusters, respectively, and several ORFs within each cluster display a significant degree of similarity to the *hrp/hrc* cluster of pathogenic bacteria. Following the nomenclature initially proposed by Preston and co-workers [112] for the *type three secretion* (*tts*) gene cluster of *P. fluorescens* SBW25, we named these genes *rsp* (rhizosphere-expressed secretion protein) or *rsc* (rsp conserved). No *tts* gene cluster or individual ORFs encoding T3SS components could be identified in the WCS358 genome, suggesting that this strain lacks a typical T3SS. Immediately adjacent to the *rspL* regulatory gene of the WCS417 *tts* cluster, a large ORF is present that encodes RopE of the AvrE family of effectors. Effectors of the AvrE family can suppress the plant’s basal immune responses and promote cell death of the host [113]. To identify additional type III effectors in the WCS417 and WCS374 genomes, we employed bioinformatical analyses searching for conserved Hrp (Rsp) “box motifs” in the promoter regions of putative effectors and exploring the N-terminal protein sequence of candidate effectors for features typical of type III secreted effectors (i.e. abundance of Ser and polar residues, acidic residues in the first 12 positions, and an aliphatic amino acid in position 3 or 4) [114, 115]. Based on these criteria, we identified 11 putative effectors for WCS417 and 15 putative effectors for WCS374 (Additional file 8: Table S3), whereas not a single putative effector could be identified in WCS358. Interestingly, the majority of WCS417 and WCS374 effectors show no homology to known effector families (data not shown), suggesting that these proteins represent novel effectors.

Although recently the genes for a Type IV secretion system (T4SS) were found in a *Pseudomonas* genome [61], T4SSs are uncommon in *Pseudomonas spp.* and were not detected in the WCS strains.

The type V secretion system (T5SS) is the most simple of all Gram-negative bacterial secretion systems as the secreted proteins are transported across the outer membrane with the aid of a covalently connected translocator domain (autotransporters) or via a single dedicated outer membrane protein (called two-partner secretion or TPS) [104]. The C-terminal translocator domain of T5SS (PFAM03797) is conserved among different classical monomeric autotransporters (T5aSS). In total, we found 2, 10, and 5 T5aSS autotransporters with this domain in WCS358, WCS374, and WCS417, respectively (Additional file 6: Table S2). Although the functions of these autotransporters are unclear, some are homologous to autotransporters of *P. aeruginosa* PAO1 or other mammalian pathogens with functions in host immune activation [116, 117], peptidase activity [118], or biofilm formation and motility [119, 120].

In addition to the T5aSS, the WCS genomes were mined for orthologs of ORFs PA0692, PA4540 and PA4624, which code for the outer membrane components of the T5bSS two-partner secretion systems in *P. aeruginosa* PAO1. Nine orthologs were found in the WCS genomes: 2 in WCS358, 4 in WCS374, and 3 in WCS417. The orthologs found in this way all contained a periplasmic polypeptide transport-associated (POTRA) domain required for recognition of the substrate protein TpsA that is transported by the T5bSS. Although significant orthologs for *P. aeruginosa* PAO1 *tpsA* genes could not be found in the WCS genomes, six putative *tpsA* genes could be identified that contained the “haemagglutinin activity domain” (pfam05860), which specifically interacts with the POTRA domains in the TpsB component [104]. The *tpsA* genes were found in the same operon and adjacent to a *tpsB* gene that is likely dedicated to its secretion. Many TpsAs are toxins that play a role in contact-dependent growth inhibition of neighboring bacteria [121]. A clear example is PD374_00815, which carries a DUF637 and a pre-toxin Hint (PT-Hint) domain, which are characteristic for such toxic TpsAs. It is therefore likely that TpsAs enable the WCS strains to inhibit their competitors and successfully colonize the plant roots. For three of the putative TpsBs, no TpsA component could be detected. To our knowledge, such stand-alone TpsB proteins have not been previously described.

Using YadA and invasin of *Yersinia enterocolitica* as bait in BLAST searches, we did not detect members of the trimeric autotransporters (T5cSS) or intimin/invasin family (T5eSS) in the WCS strains. However, in each of the WCS genomes, a single ortholog of Patatin-like protein PlpD of *P. aeruginosa* PAO1 was found, which was recently suggested to represent a novel T5SS (T5dSS) [121]. Patatins form a group of glycoproteins with lipolytic activity found in potato tubers and have proposed functions against plant pathogens [104, 122, 123].

Type VI secretion systems (T6SS) are functionally similar to T3SS in that they deliver effector-like proteins into other organisms. Therefore, like T3SS, T6SS have been implied to play a role in manipulation of host immunity. They also play a prominent role in bacterial warfare by delivering toxic proteins into competing micro-organisms [124]. The machinery structurally resembles the contractile tails of bacteriophages and is used, in this case, for injecting toxins into target cells. Protein sequences corresponding to the genes encoded in the T6SS loci of *P. aeruginosa* PAO1 were used as bait in BLASTp searches of the three WCS genomes. Clusters containing five or more genes with significant similarity to the bait genes (e-value < 10^−5^) were considered a T6SS locus [125]. The genomes of WCS417 and WCS374 each contained two T6SS loci, while WCS358 contained one.

Overall, these data display the diversity of the protein secretion systems in WCS358, WCS374, and WCS417. Their presence is not surprising, but highlight the sophisticated mechanisms that these plant-beneficial rhizosphere bacteria have evolved in order to successfully compete with other soil microbiota and sustain a long-term mutualistic relationship with their host plants.

## Discussion

**Fig. 7:**
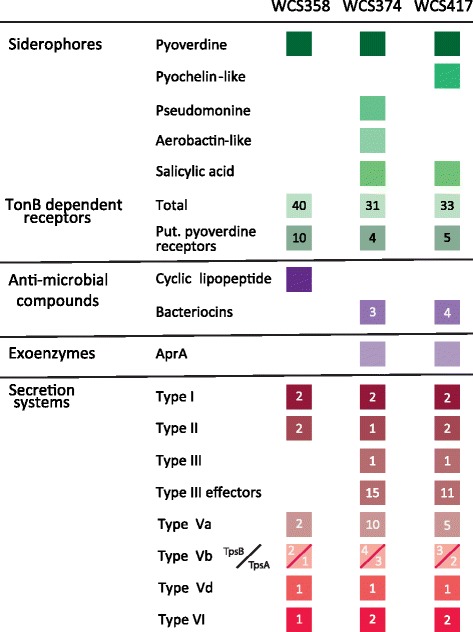
Selected rhizosphere competence traits in WCS strains. Overview of selected traits that contribute to rhizosphere competence of WCS358, WCS374, and WCS417. Traits are divided in groups related to siderophore-mediated competition for iron, antimicrobial compound production, exoenzymes, and protein secretion systems. Colored boxes indicate the presence of a gene or gene cluster. Absence of a box means absence of the gene or gene cluster. Numbers within boxes indicate the number of copies of genes or gene clusters in the corresponding genome

In the past 30 years, the plant-beneficial *Pseudomonas* model strains WCS358, WCS374 and WCS417 have been extensively studied for their plant-protective traits. In this study, we explored their genomes and established an overview of their phylogeny and traits supporting rhizosphere competence and plant functioning (Fig. 7).

### WCS strains are representatives of universally-present plant-beneficial *Pseudomonas* spp.

The genus *Pseudomonas* is very diverse and its taxonomy is still subjected to refinement [53]. Ideally, the taxonomy of *Pseudomonas* spp. reflects their evolutionary history and is predictive for their functionality [126]. Previously, WCS358 was classified as *P. putida* and both WCS374 and WCS417 as *P. fluorescens* [54, 55]. Although these WCS strains are indeed closely related to the *P. putida* and *P. fluorescens* type strains, our genome analysis revealed that the level of similarity is not sufficiently high to group the WCS strains within these species. Instead, WCS417 was found to be very closely related to the *P. simiae* type strain and can be regarded as a representative of this species. The level of sequence homology of WCS358 and WCS374 did not fall within the boundary of any other *Pseudomonas* type strain. Hence, WCS358 and WCS374 should be regarded as a yet undescribed species. Genome information from *Pseudomonas* strains A506 and SS101 revealed that these strains are very similar to WCS374 and fall within the boundary of the same so far undescribed *Pseudomonas* species. WCS374, A506 and SS101 were all isolated from plants, albeit from different hosts (potato, pear and wheat, respectively), and different geographic locations in the Netherlands and Oregon [20, 52, 64, 127]. Since all three strains have been selected and studied for their abilities to protect plants against a wide range of pathogens, we propose the name *Pseudomonas defensor* for this species and strain WCS374 as its type strain. Strain WCS358 was also studied extensively because of its ability to protect plants against pathogen infection. Because the plant protective properties of WCS358 are predominantly linked to siderophore production and sequestration of iron, we propose the name *Pseudomonas capeferrum* (‘seizes iron’) for the species and WCS358 as its type strain. This species also includes *Pseudomonas* strain CFBP2461, which was isolated from roots of bean in France. Like WCS358, CFBP2461 has also been studied for its plant-protective capabilities and produces exactly the same siderophore as WCS358 [68].

For each of the WCS strains we found one or more other representatives of the same species, all of which surfaced in the *Pseudomonas* research community because of their plant-beneficial properties. The strains were isolated from different hosts and at different geographic locations, indicating that the sequenced WCS strains can be regarded as universal representatives of a group of *Pseudomonas* spp. with plant protective functions. The genomes of both WCS374 and WCS417 were so similar to the genomes of their respective closest relatives that they are nearly isogenic, even though these strains were isolated decades and continents apart. This is remarkable considering the enormous diversity of *Pseudomonas* species in the soil and the relatively small number of bacterial genomes that have been sequenced [128, 129]. The fact that different labs independently isolate very closely-related bacterial strains with similar plant protective properties at different locations across the World, suggests that plants have mechanism to specifically enrich their microbiome for such plant-beneficial microbes.

### WCS genomes support different strategies for iron acquisition

Originally, the WCS strains were studied for their remarkable capacity to control plant pathogens through siderophore-mediated competition for iron. The WCS strains have evolved different strategies for the acquisition of iron. WCS358 seems to have limited options as it can only produce one siderophore, PVD358. However, very few *Pseudomonas* strains seem to be able to use this pyoverdine. Only 1 % of over 1000 randomly isolated *Pseudomonas* strains could utilize PVD358, whereas 30 % of these strains were able to use PVD374 [33]. In addition, WCS358 is able to utilize the pyoverdines produced by a wide range of other *Pseudomonas* strains [33], which is corroborated by the large number of pyoverdine receptors found in the genome of WCS358 (Fig. 7).

By contrast, WCS374 produces a less specific pyoverdine, and is not able to use as many heterologous siderophores as WCS358. The latter is reflected by the lower number of putative pyoverdine receptor genes in the genome of WCS374 (Fig. 7). This suggests that many other bacteria are able to utilize PVD374, while in turn WCS374 is not well capable of utilizing pyoverdine of competing bacteria. However, WCS374 can produce a different siderophore, pseudomonine. The competitive success of WCS358 and WCS374 has been investigated in the rhizosphere of radish [130]. After co-inoculation, WCS358 reduced population densities of wild-type WCS374 by up to 30-fold, whereas WCS374pMR, a WCS374 derivative harboring the PVD358 receptor *pupA* of WCS358 maintained its population density. Population densities of WCS358 were unaffected by co-inoculation with WCS374 or any of 20 random radish-rhizosphere isolates. This demonstrates that pyoverdine-mediated competition for iron is a major factor in the colonization of radish roots by these strains and that the iron acquisition strategy of WCS358 is more advantageous than that of WCS374.

The iron sequestration strategy of WCS417 is intermediate to that of WCS358 and WCS374. It has fewer ferric pyoverdine receptors than WCS358, but one more than WCS374 (Fig. 7). We discovered a gene cluster in the WCS417 genome that is involved in the putative production of a pyochelin-like siderophore, indicating it produces a siderophore additional to pyoverdine. Moreover, although the WCS417 siderophore PVD417 is very similar to PVD374, the epimerization module of the NRPS involved in biosynthesis of PVD417 is predicted to modify the configuration of one of the six amino acid residues in the peptide chain of PVD417. It is tempting to speculate that this is an evolutionary adjustment to decrease the number of other bacteria that are capable of using PVD417.

### ISR elicitors encoded by the WCS genomes

The production of pyoverdines has been demonstrated to play an important role in the biological control activity of the WCS strains in the rhizosphere. Flax, potato, radish, eucalypt, carnation, bean and tomato were less diseased when their roots were colonized by one of the WCS strains, while this protection was no longer observed when pyoverdine knockout derivatives of the strains colonized the roots of these plants [22, 25, 27, 28, 44, 75, 131]. However, this protective effect is not always solely based on siderophore-mediated competition for iron, as pyoverdines were also implicated in the onset of ISR. PVD358 has been demonstrated to elicit ISR in Arabidopsis, bean, eucalypt and tomato [44, 131]. In tobacco, PVD358, PVD374 and PVD417 have all been demonstrated to trigger ISR [132]. In radish, only PVD374 is an effective ISR elicitor, whereas PVD358 and PVD417 are not [25]. This suggests that siderophores may play a role in host specificity with respect to the ability of the rhizobacterial strains to elicit ISR. Interestingly, our genome analysis predicts that PVD374 and PVD417 only differ in the isomeric configuration of one of the amino acids incorporated in the otherwise-identical peptide chain suggesting that plants can distinguish such minute differences between pyoverdines.

Besides pyoverdine, WCS374 also produces the siderophore pseudomonine, whereas the other WCS strains do not (Fig. 7). Interestingly, pseudomonine has a SA moiety in its molecule, the biosynthesis of which is encoded by the isochorismate synthase gene *pmsC* and the isochorismate-pyruvate lyase gene *pmsB.* In WCS374, both genes are part of the pseudomonine biosynthesis gene cluster *pmsCEAB* [76]. SA has been demonstrated to be a potent inducer of systemic acquired resistance (SAR). However, while exogenously applied SA induces systemic disease resistance in Arabidopsis, the SA producer WCS374 does not [48]. It was proposed that during colonization of plant roots by WC374, SA becomes swiftly incorporated in the siderophore pseudomonine, thereby preventing free SA from acting as an elicitor of plant immunity [70]. Interestingly, the close relative of WCS374, *Pseudomonas* strain SS101, is capable of inducing a systemic resistance in Arabidopsis, and it does so in an SA-dependent manner [133]. The SA biosynthesis genes of SS101 are highly similar to those of WCS374, but it may be possible that SS101 produces more SA in the rhizosphere or incorporates it less efficiently in its pseudomonine, thereby allowing the activation of the SAR pathway.

The nature of other potential ISR elicitors produced by the WCS strains has most extensively been investigated for WCS358 [44]. In Arabidopsis, purified pyoverdine, LPS and flagella of WCS358 were all demonstrated to be able to trigger ISR. Interestingly, knockout mutants of WCS358 that no longer produced one of these determinants also triggered ISR, which was concluded to be a logical consequence of the redundancy of these determinants. In tomato, the cyclic lipopeptide massetolide A of SS101 emerged as an additional elicitor of ISR [96]. The cyclic lipopeptide predicted to be produced by WCS358 might similarly be involved in ISR. Cyclic lipopeptides are also important in rhizosphere competence as they can inhibit other microbes and influence biofilm formation [71]. In this study, we were able to gain insight into the nature of the biosynthetic genes of all these ISR elicitors, which will be highly instrumental in future studies on the molecular mechanisms of the onset of ISR by plant-beneficial rhizosphere bacteria.

### Protein secretion systems encoded by the WCS genomes in relation to rhizosphere competence

To protect plants from harmful organisms, PGPR need to reach sufficiently high population levels in the rhizosphere. For siderophore-mediated competition for iron or the onset of ISR, cell densities of at least 10^5^ per gram of root have to be reached by the WCS strains [26]. Similar threshold densities have been reported for other PGPR [134, 135]. This requires that the PGPR strains are capable of out competing other microorganisms. To date, six types of protein secretion systems are recognized in bacteria, most of which play a role in host-microbe and microbe-microbe interactions. The Type I, II, III, V, and VI secretion systems were represented in the genomes of all three WCS strains (Fig. 7). The T1SSs and T2SSs of the WCS are likely to be involved in the secretion of extracellular enzymes, like extracellular protease AprA, lipase and phosphatase to facilitate nutrient acquisition [108, 134]. In addition, T1SS and T2SS may facilitate the secretion of antimicrobial compounds, such as cyclic lipopeptides [71], or bacteriocins [101] for which we identified biosynthetic genes in the genomes of the WCS strains. The genomes of WCS374 and WCS417 possess a gene cluster that encodes a putative T3SSs, as well as several genes encoding so far unknown putative effector proteins. T3SSs have been demonstrated to be functional in strains of the *P. fluorescens* group [112, 136, 137, 138]. However, their ecological significance in the rhizosphere is still unclear, as *t3ss* mutants of several *Pseudomonas* strains are not hampered in their ability to retain high population levels in the rhizosphere [112, 137, 139]. For the T5SS, a wide diversity of T5SS-related genes were identified in the WCS genomes. Many proteins that are secreted via the T5bSS are toxins that can play a role in contact-dependent growth inhibition of neighboring bacteria [121], thereby contributing to rhizosphere competence of the WCS strains. The same holds true for the T6SSs that were identified in the WCS genomes. T6SS have been demonstrated to function in competition with other microbes by delivering bactericidal effectors in a cell-contact dependent manner [103, 140, 141].

### Suppression of root immune responses

To establish a long-lasting mutual relationship with their hosts, mutualistic microbes need to suppress or evade local host immune responses that are triggered in plant roots upon recognition of alien organisms [138]. For WCS417 it has been demonstrated that colonization of Arabidopsis roots by this rhizobacterium suppresses local root immune responses that are typically triggered by the flagellin epitope flg22 [50]. A possible mechanism by which WCS417 prevents the activation of this innate immune response is through the production of the alkaline protease AprA. AprA of *P. aeruginosa* and *P. syringae* was recently demonstrated to degrade flagellin monomers, thereby preventing flg22-triggered immunity in the leaves of Arabidopsis [142, 143]. The *aprA* gene found in the T1SS operon of the WCS417 genome will be instrumental in addressing this question. Another way by which plant-beneficial *Pseudomonas* strains may suppress local host immune responses is via T3SS-mediated injection of effector proteins. Pathogenic bacteria utilize this specialized secretion machinery to inject effector molecules into the cytoplasm of eukaryotic cells in order to suppress host immune responses and establish successful infections. In several non-pathogenic root-associated *Pseudomonas* bacteria, functional T3SSs or T3SS gene clusters have been found [52, 112, 137]. The T3SSs and putative effector proteins identified in WCS374 and WCS417 and the fact that we did not find T3SS-related genes in the genome of WCS358 may prove to be instrumental in investigating the role of T3SS-related effectors in suppression of root immunity.

## Conclusions

In the past 30 years, a wealth of knowledge accumulated on the plant-beneficial functions of the PGPR WCS358, WCS374 and WCS417. The fully sequenced genomes of the WCS strains provide a genetic framework that allows for detailed analysis of the biological mechanisms of the plant-beneficial traits of the PGPR. The WCS genomes also clarified their taxonomy and revealed that very similar strains from the same species have been isolated elsewhere in the World because of their plant-beneficial properties. Hence, the sequenced WCS strains can be considered representatives of universally-present plant-beneficial *Pseudomonas* spp. with well-characterized functions in the stimulation of plant growth and health. Considering the increasing focus on the role of the root microbiome in plant health, functional genomics of the WCS strains will foster research toward understanding the diversity of functions of the root microbiome.

## Methods

### Sequencing, genome assembly, and annotation

The genomes of *Pseudomonas* spp. strains WCS358, WCS374, and WCS417 were sequenced following a whole-genome shotgun strategy using an Illumina HiSeq 2000 and two paired-end libraries with 500- and 2000-bp insert size at the Beijing Genomics Institute (BGI; Shenzhen, China). A hybrid genome assembly was prepared using SOAPdenovo (version 1.05) and ABySS (version 1.3.4). Multiple gaps were closed by merging overlapping contigs. The order of the remaining contigs was determined by alignment to close taxonomic relatives with MAUVE. Contigs could be combined in a single scaffold for WCS417 and WCS374. For WCS358, the order of the remaining contigs could not be determined as it differed depending on the relative it was compared to. The genome of WCS358 therefore remained distributed over eight scaffolds. Genome sequences were deposited at Genbank under the accessions CP007637 (WCS417), CP007638 (WCS374) and JMIT00000000 (WCS358). The genomes were annotated using NCBI’s Prokaryotic Genome Automatic Annotation Pipeline [144].

### Bioinformatic analyses

Concatenated sequences of *16S rRNA*, *gyrB, rpoB* and *rpoD* genes of WCS358, WCS374, and WCS417 were compared to the corresponding sequences of all *Pseudomonas* spp. type strains as described by Mulet *et al.* [53], and to the corresponding genes of plant-beneficial members of the *P. fluorescens* group of which the full genome was available [52, 145, 146]. *16S rRNA*, *gyrB, rpoB* and *rpoD* genes of *Pseudomonas* sp. CFBP 2461 were amplified as described by Mulet *et al.* [53]. Throughout this study, alignments and phylogenetic trees were created with CLC main Workbench 6.7.2 (CLCbio, Aarhus, Denmark) using the neighbor joining algorithm and 1000 bootstrap replicates. Average Nucleotide Identity based on BLAST (ANIb) values were calculated using Jspecies [62]. Multiple genome alignments were performed with progressive MAUVE [147]. Genomic islands were identified using Islandviewer [65].

Mining for orthologs of genes in the genomes of WCS358, WCS374, and WCS417 was performed using reciprocal BLASTp analysis. Genes were considered orthologs when e-value <10^−5^. Characteristic protein domains were identified using the Pfam protein families database [148].

The genomes were screened for secondary metabolite biosynthetic clusters using AntiSMASH 2.0 [149]. Bacteriocin biosynthesis clusters were examined with BAGEL3 [102].

The amino acid composition of non-ribosomal peptide synthetase (NRPS) products was predicted using AntiSMASH, the PKS/NRPS Analysis Web-site [150] and the NRPS predictor 2 [151].

### Cultivation of bacteria and media

The *Pseudomonas* spp. strains used in this study are listed in Table 3. All bacteria were routinely cultivated on King’s medium B agar (KBA; [152]) at 28 °C. Production of surfactants was tested in a drop-collapse assay as described by Kuiper *et al.* [100]. Production of siderophores by the WCS strains and their mutant derivatives was tested on CAS agar [73]. The abilities of the WCS strains and their mutant derivatives to sequester iron was tested by inoculating 10 μl of bacterial suspension (OD_660_ of 0.1) in 10 mM MgSO_4_ on KBA amended with a range of 2,2-bipyridyl concentrations (0, 400, 600, 800, 1000, 1200, 1400 or 2000 μM). Bacterial growth was assessed after 24 h of incubation at 28 °C. Cross feeding of *P. fluorescens* strain Pf-5 *fpv* mutants by the WCS strains was examined as described by Hartney *et al.* [32] on KBA. Briefly, bacterial cells were suspended in 10 mM MgSO_4_ at OD_660_ of 0.1 for the WCS strains and 10^−2^ dilutions thereof for the Pf-5 derivatives. Droplets of 10 μl were placed on the surface of KBA amended with 600 μM 2,2-bipyridyl. Receptor mutants were placed 1 cm apart from the WCS strains and growth of receptor mutants was examined after 48 h of incubation at 28 °C. Protease activity was assessed as described by Pel and co-workers [142] on KBA containing skimmed milk powder.

**Table 3:** Biocontrol strains and their mutant derivatives that were used in this study

Strain	Relevant characteristics	References
*Pseudomonas capeferrum* WCS358r	Spontaneous rifampicin-resistant mutant of WCS358. Wild type isolated from potato rhizosphere. Produces siderophore PVD358^1^	[20]
WCS358-PVD^−^	Tn*5* mutant of WCS358r that does not produce PVD358; Original strain name WCS358-JM213.	[74]
*Pseudomonas* defensor WCS374r	Spontaneous rifampicin-resistant mutant of WCS374. Wild type isolated from potato rhizosphere. Produces siderophores PVD374 and PSM374^2^, and the PSM precursor SA^3^	[20]
WCS374- PVD^−^	PVD^−^, PSM^+^, SA^+^, Tn*5* transposon mutant of WCS374r that does not produce PVD374. Original strain name WCS374-02.	[156]
WCS374- PMS^−^	PVD^+^, PSM^−^, SA^+^, *pmsA* mutant^4^ of WCS374r obtained by site-directed mutagenesis. Original strain name WCS374-4A1.	[24, 70]
WCS374- PVD^−^ PSM^−^	PVD^−^, PSM^−^, SA^+^, Tn*5* transposon mutant of WCS374-4A1 that produces neither PVD374 nor Pseudomonine. Original strain name WCS374-AT12.	[24, 70]
WCS374-PSM^−^ SA^−^	PVD^+^, PSM^−^, SA^−^, *pmsB* mutant^5^ of WCS374r obtained by site-directed mutagenesis. Original strain name WCS374-4B1.	[24, 70]
WCS374-PVD^−^ PSM^−^-SA^−^	PVD^−^, PSM^−^, SA^−^, Tn*5* transposon mutant of WCS374-4B1 that does not produce PVD374. Original strain name WCS374-BT1.	[24, 70]
*Pseudomonas simiae* WCS417r	Spontaneous rifampicin-resistant mutant of WCS417. Wild type was isolated from wheat rhizosphere. Produces siderophore PVD417	[21, 48]
WCS417-PVD^−^	Tn*5* mutant of WCS417 that does not produce PVD417. Original strain name WCS417-M634.	[75]
*Pseudomonas capeferrum* CFBP 2461	Wild-type isolated from bean rhizosphere; produces PVD358	[67]
*P. protegens* Pf-5	Wild-type isolated from cotton rhizosphere; produces PVD-Pf-5 and enantio-pyochelin	[157]
LK032	Mutant derivative of PF-5 Δ*pchA* ^*6*^Δ*pvdI* ^*7*^ *.* Does not produce siderophores.	[32]
LK148	Mutant derivative of LK032 Δ*pchA*Δ*pvdI*Δ*fpvY.* Does not produce siderophores and defective in ferric-pyverdine receptor FpvY	[32]
LK150	Mutant derivative of LK032 Δ*pchA*Δ*pvdI*Δ*fpvX.* Does not produce siderophores and defective in ferric-pyverdine receptor FpvX	[32]
LK151	Mutant derivative of LK032 Δ*pchA*Δ*pvdI*Δ*fpvV.* Does not produce siderophores and defective in ferric-pyverdine receptor FpvV	[32]
LK153	Mutant derivative of LK032 Δ*pchA*Δ*pvdI*Δ*fpvW.* Does not produce siderophores and defective in ferric-pyverdine receptor FpvW	[32]
LK154	Mutant derivative of LK032 Δ*pchA*Δ*pvdI*Δ*fpvU* . Does not produce siderophores and defective in ferric-pyverdine receptor FpvU	[32]
LK155	Mutant derivative of LK032 Δ*pchA*Δ*pvdI*Δ*fpvZ.* Does not produce siderophores and defective in ferric-pyverdine receptor FpvZ	[32]

^1^PVD, siderophore pyoverdine

^2^PSM, siderophore pseudomomine

^3^SA, salicylic acid, precursor of pseudomonine and pyochelin

^4^ *pmsA*, gene involved in the synthesis of the histamine moiety of Pseudomonine

^5^ *pmsB,* gene involved in the biosynthesis of SA as a precursor of Pseudomonine

^6^ *pchA* gene encoding an isochorismate synthetase involved in production of SA/pyochelin

^7^ *pvdI* geneencoding a non-ribosomal peptide synthase involved in production of pyoverdine

## Availability of supporting data

The genomes sequenced in this study are available at the website of the National Center for Biotechnology Information:

http://www.ncbi.nlm.nih.gov/nuccore/CP007637.1

http://www.ncbi.nlm.nih.gov/nuccore/CP007638.1

http://www.ncbi.nlm.nih.gov/nuccore/NZ_JMIT00000000.1

All other supporting data are included as additional files

## Additional files

Additional file 1: Figure S1. Venn diagrams showing the number of unique genes and genes shared between A) Pseudomonas strain WCS417 and R81 and between B) Pseudomonas strains WCS374, A506 and SS101.

Additional file 2: Table S1. Lists of unique genes of *Pseudomonas* strain WCS374, not present in A506 and SS101 and vice versa and of WCS417 not present in R81 and vice versa.

Additional file 3: Figure S2. Phylogenetic analysis of pyoverdine receptor genes in WCS358, WCS374, and WCS417. Neighbor-joining tree of all TonB-dependent proteins (TBDPs) identified in the WCS genomes and six confirmed ferric-pyoverdine receptors (FPVs) identified in the genome of *P. protegens* Pf-5 (in blue font). TBDPs with an N-terminal signaling domain characteristic of TonB-dependent transducers are indicated in green font; TBDPs without this domain are in red font. Bootstrap values from 1000 replicates are indicated at the nodes. Some of the WCS TBDPs clustered closely together with the FPVs of Pf-5, which indicates they have the same substrate. FpvU and FpvY, responsible for the uptake of PVD374 in Pf-5, clustered together with TBDP PC358-17710, PD374_15530 and PS417_11700 indicating that these are the receptors used for the uptake of PVD417 and PVD374 by the three strains. Previously described FPVs of WCS358 were among the 10 putative FPVs of WCS358 in the tree. PupA, which is required for the uptake of PVD358 [158, 159], does not seem to have a closely related FPV in the three other strains, which concurs with the fact that none of the other strains can be cross-fed by WCS358 on iron-limited medium. Again this receptor for PVD358 was found in the operon responsible for the biosynthesis of its peptide chain and immediately adjacent to the last NRPS gene. PupB clustered with the FpvV of Pf-5 and both TBDPs are responsible for the uptake of PVDBN7 of BN7 [160, 161]. Likewise, the TBDP RF3 clustered together with FpvW and both were demonstrated to function in the uptake of PVDB10 of B10. Although, RF2 and FpvZ clustered together, they are likely to have different substrates [32, 160]. This indicates that for definite conclusions on the substrates of the TBDPs found in this *in silico* analysis, further *in vivo* confirmation is required.

Additional file 4: Figure S3. Identification of Pf-5 siderophore receptors involved in heterologous uptake of siderophores of WCS358, WCS374, and WCS417. Cross feeding of *P. protegens* Pf-5 and its siderophore receptor mutant derivatives LK032, LK148, LK150, LK151, LK153, LK154 and LK155 by siderophore donor strains WCS358, WCS374 and WCS417. WCS strains were placed in the center of the KBA plate that was supplemented with 600 μM 2,2-bipyridyl to create conditions of low iron availability. Letters indicate the TonB-dependent protein (TBDP) siderophore receptor mutants of Pf-5: A) LK032 (ΔpchA ΔpvdI), B) LK148 (ΔpchA ΔpvdI ΔfpvY), C), LK150 (ΔpchA ΔpvdI ΔfpvX), D) LK151 (ΔpchA ΔpvdI ΔfpvV), E) LK153 (ΔpchA ΔpvdI ΔfpvW), F) LK154 (ΔpchA ΔpvdI ΔfpvU), and G) LK155 (ΔpchA ΔpvdI ΔfpvZ). This set of mutants all lack biosynthesis genes for the siderophores pyochelin (ΔpchA) and pyoverdin (ΔpvdI), allowing to differentially test for the requirement of the TBDP receptors FpvU to FpvZ for the uptake of WCS siderophores.

Additional file 5: Figure S4. Drop collapse assay for surfactant production by WCS strains. Bacterial cells of the WCS strains were suspended in a droplet of water placed on parafilm. Collapse of the droplet is an indication of surfactant production.

Additional file 6: Table S2. Genes of WCS358, WCS374, and WCS417 putatively involved in protein secretion systems type I, II, III, IV, and VI. Gene clusters involved in protein secretion were identified in a BLASTp search with protein sequences of gene clusters of secretion systems in *P. aeruginosa*.

Additional file 7: Figure S5. Protease activity of *Pseudomonas* strains on milk plates. *Pseudomonas syringae* pathovar tomato DC3000, its AprA-defective derivative [142] and the WCS strains WCS358, WCS374 and WCS417 were grown on KBA amended with skimmed milk powder. A clearing zone around the colony indicates extracellular protease activity.

Additional file 8: Table S3. List of putative type III secreted effectors. Putative type III secreted effector genes were identified by searching for conserved Hrp (Rsp) “box motifs” in the promoter regions of putative effectors and exploring the N-terminal protein sequence of candidate effectors for features typical of type III secreted effectors (i.e. abundance of Ser and polar residues, acidic residues in the first 12 positions, and an aliphatic amino acid in position 3 or 4) [114, 115].

## Abbreviations

ANIb: Average nucleotide identity based on BLAST
ET: Ethylene
FPV: Ferric-pyoverdine receptors
ISR: Induced systemic resistance
JA: Jasmonic acid
LPS: Lipopolysaccharides
MAMP: Microbe-associated molecular patterns
MLSA: Multi-locus sequence alignement
NI: Nucleotide identity
NRPS: Non-ribosomal peptide synthethase
OBL: O-antigen biosynthetic loci
PGPR: Plant-growth promoting rhizobacteria
PSM: Pseudomonine
PVD: Pyoverdine
SA: Salicylic acid
SAR: Systemic acquired resistance
T1SS, T2SS, etc.: Type I secretion system, Type II secretion system, etc.
TBDP: TonB dependent prtoeins
WCS: Willie Commelin Scholten
