# Supplementary figures and images for: Unearthing the genomes of plant-beneficial Pseudomonas model strains WCS358, WCS374 and WCS417
Source: BMC Genomics. 2015 Jul 22;16(1):539. doi: 10.1186/s12864-015-1632-z (PMC4509608; doi:10.1186/s12864-015-1632-z)

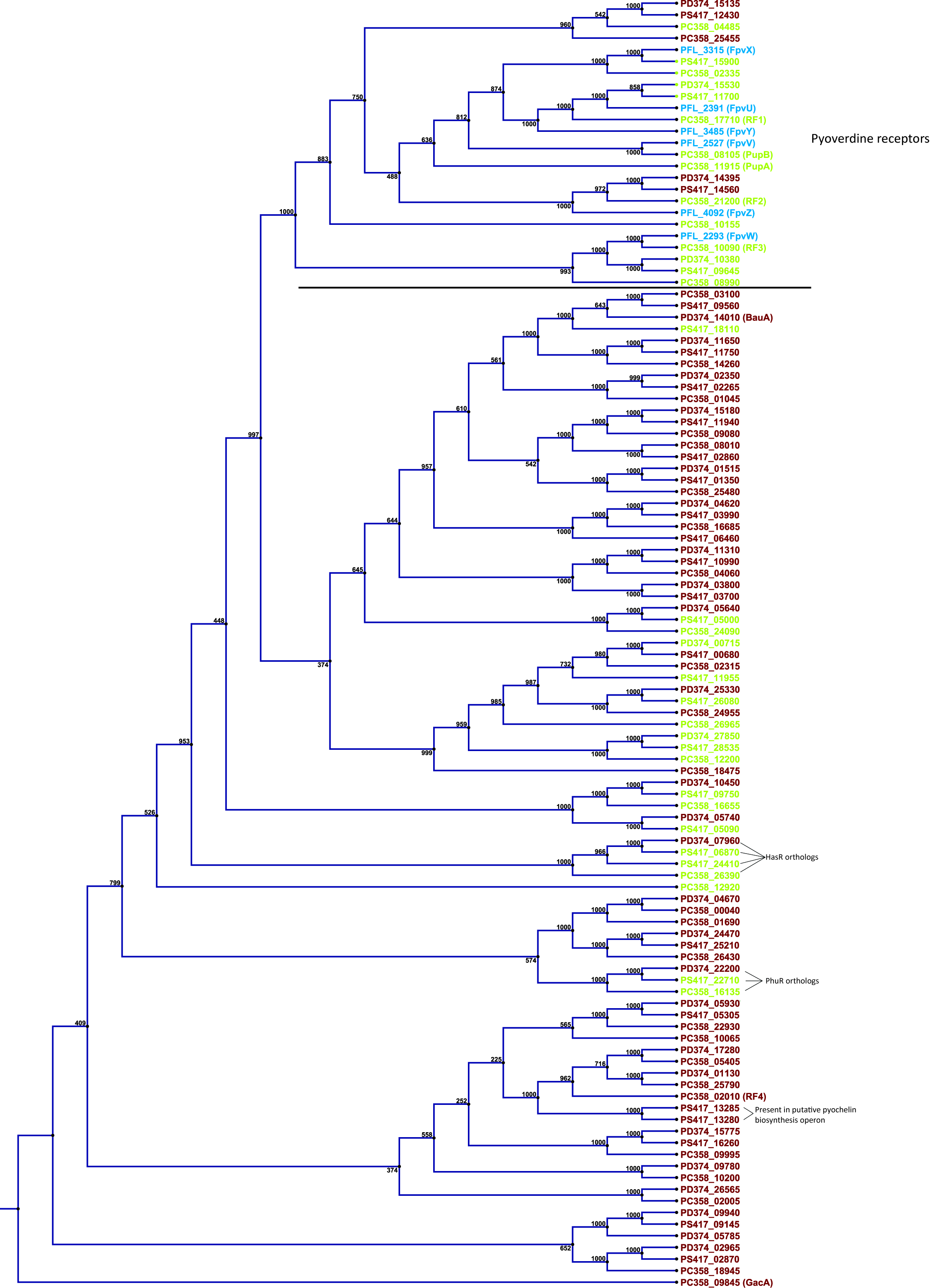

Supplement: Supplementary file 3 — Phylogenetic analysis of pyoverdine receptor genes in WCS358, WCS374, and WCS417. Neighbor-joining tree of all TonB-dependent proteins (TBDPs) identified in the WCS genomes and six confirmed ferric-pyoverdine receptors (FPVs) identified in the genome of P. protegens Pf-5 (in blue font). TBDPs with an N-terminal signaling domain characteristic of TonB-dependent transducers are indicated in green font; TBDPs without this domain are in red font. Bootstrap values from 1000 replicates are indicated at the nodes. Some of the WCS TBDPs clustered closely together with the FPVs of Pf-5, which indicates they have the same substrate. FpvU and FpvY, responsible for the uptake of PVD374 in Pf-5, clustered together with TBDP PC358-17710, PD374_15530 and PS417_11700 indicating that these are the receptors used for the uptake of PVD417 and PVD374 by the three strains. Previously described FPVs of WCS358 were among the 10 putative FPVs of WCS358 in the tree. PupA, which is required for the uptake of PVD358 [158, 159], does not seem to have a closely related FPV in the three other strains, which concurs with the fact that none of the other strains can be cross-fed by WCS358 on iron-limited medium. Again this receptor for PVD358 was found in the operon responsible for the biosynthesis of its peptide chain and immediately adjacent to the last NRPS gene. PupB clustered with the FpvV of Pf-5 and both TBDPs are responsible for the uptake of PVDBN7 of BN7 [160, 161]. Likewise, the TBDP RF3 clustered together with FpvW and both were demonstrated to function in the uptake of PVDB10 of B10. Although, RF2 and FpvZ clustered together, they are likely to have different substrates [32, 160]. This indicates that for definite conclusions on the substrates of the TBDPs found in this in silico analysis, further in vivo confirmation is required. [file 12864_2015_1632_MOESM3_ESM.tiff]

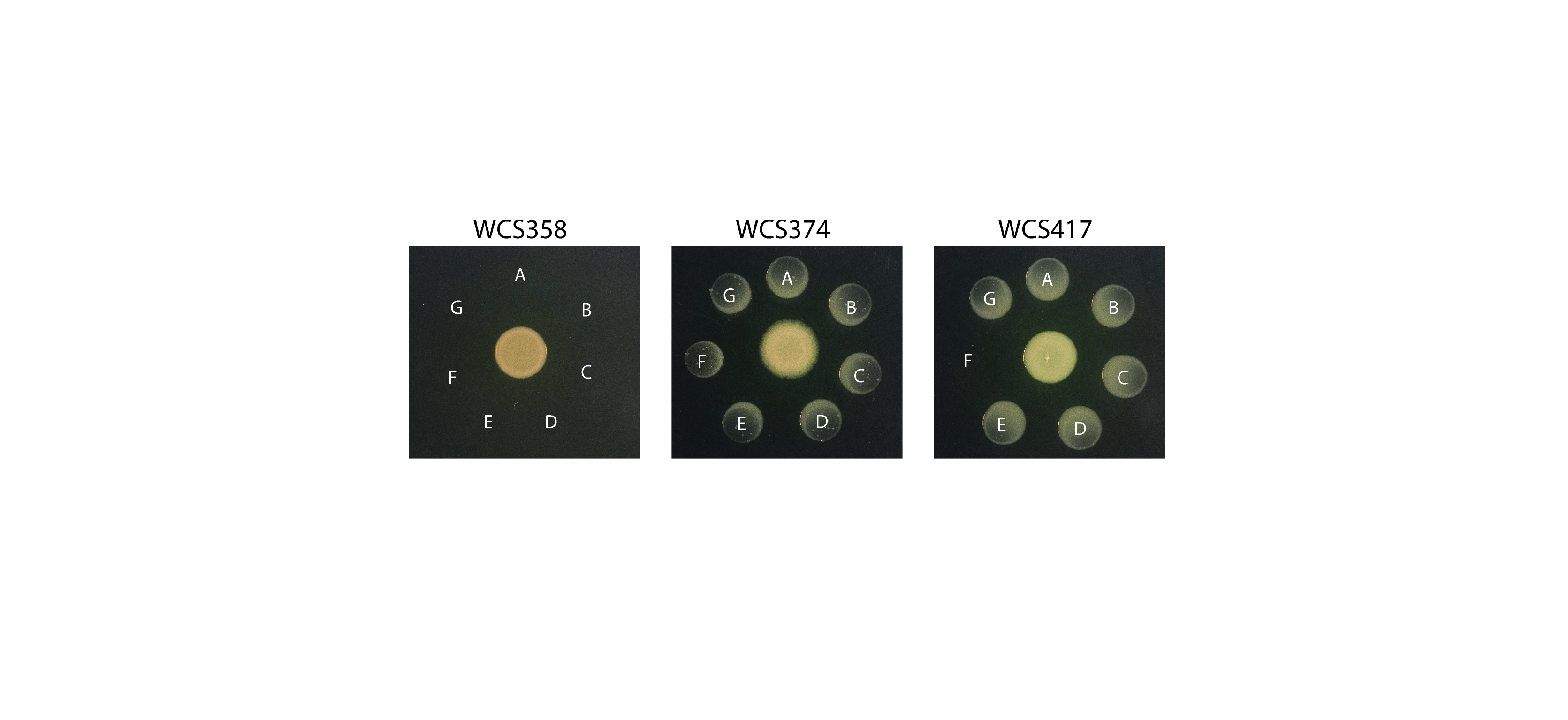

Supplement: Supplementary file 4 — Identification of Pf-5 siderophore receptors involved in heterologous uptake of siderophores of WCS358, WCS374, and WCS417. Cross feeding of P. protegens Pf-5 and its siderophore receptor mutant derivatives LK032, LK148, LK150, LK151, LK153, LK154 and LK155 by siderophore donor strains WCS358, WCS374 and WCS417. WCS strains were placed in the center of the KBA plate that was supplemented with 600 μM 2,2-bipyridyl to create conditions of low iron availability. Letters indicate the TonB-dependent protein (TBDP) siderophore receptor mutants of Pf-5: A) LK032 (ΔpchA ΔpvdI), B) LK148 (ΔpchA ΔpvdI ΔfpvY), C), LK150 (ΔpchA ΔpvdI ΔfpvX), D) LK151 (ΔpchA ΔpvdI ΔfpvV), E) LK153 (ΔpchA ΔpvdI ΔfpvW), F) LK154 (ΔpchA ΔpvdI ΔfpvU), and G) LK155 (ΔpchA ΔpvdI ΔfpvZ). This set of mutants all lack biosynthesis genes for the siderophores pyochelin (ΔpchA) and pyoverdin (ΔpvdI), allowing to differentially test for the requirement of the TBDP receptors FpvU to FpvZ for the uptake of WCS siderophores. [file 12864_2015_1632_MOESM4_ESM.tiff]

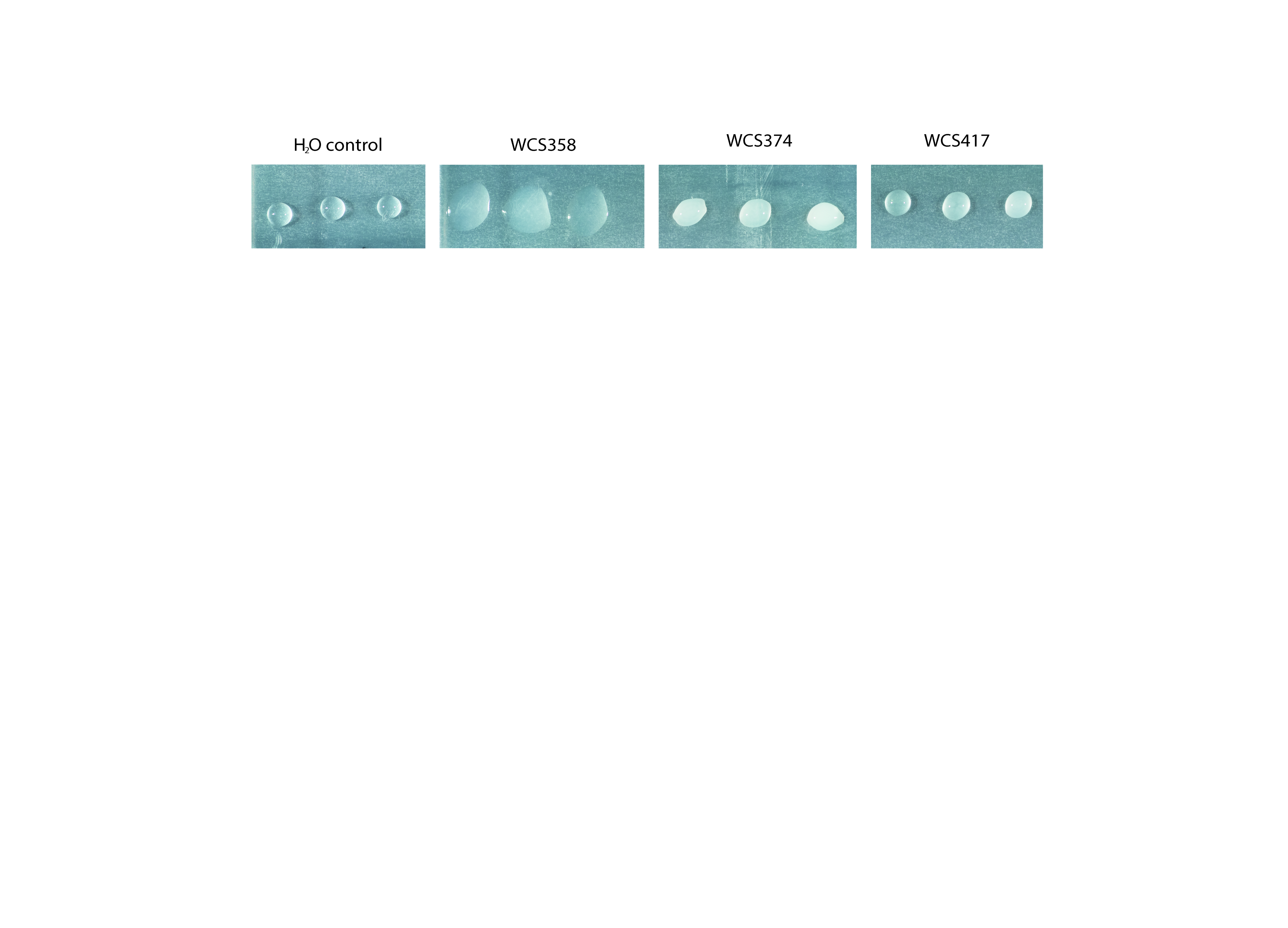

Supplement: Supplementary file 5 — Drop collapse assay for surfactant production by WCS strains. Bacterial cells of the WCS strains were suspended in a droplet of water placed on parafilm. Collapse of the droplet is an indication of surfactant production. [file 12864_2015_1632_MOESM5_ESM.tiff]

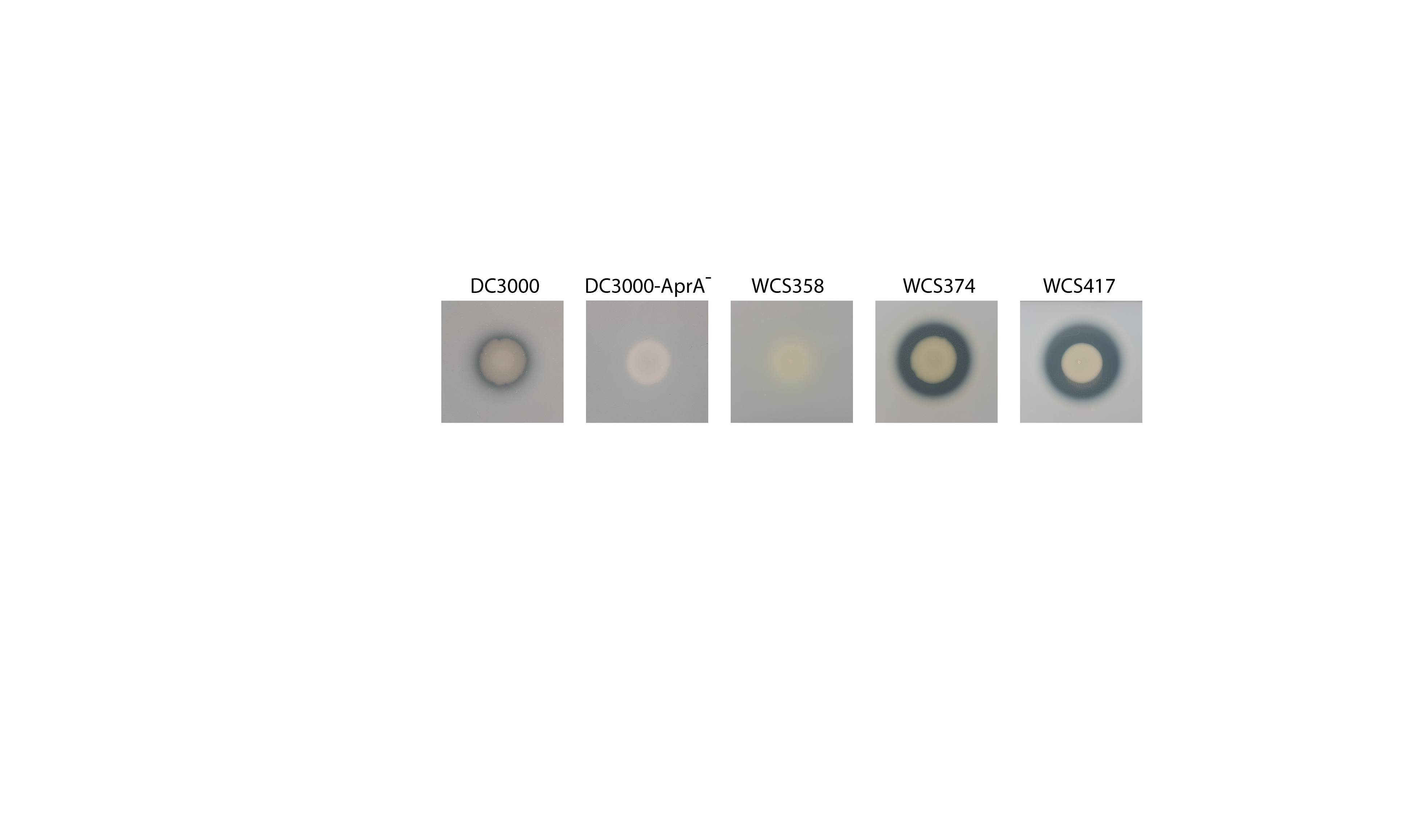

Supplement: Supplementary file 7 — Protease activity of Pseudomonas strains on milk plates. Pseudomonas syringae pathovar tomato DC3000, its AprA-defective derivative [142] and the WCS strains WCS358, WCS374 and WCS417 were grown on KBA amended with skimmed milk powder. A clearing zone around the colony indicates extracellular protease activity. [file 12864_2015_1632_MOESM7_ESM.tiff]
