# Supplementary material for: Unearthing the genomes of plant-beneficial Pseudomonas model strains WCS358, WCS374 and WCS417
Source: BMC Genomics. 2015 Jul 22;16(1):539. doi: 10.1186/s12864-015-1632-z (PMC4509608; doi:10.1186/s12864-015-1632-z)
Supplement: Supplementary file 6 — Genes of WCS358, WCS374, and WCS417 putatively involved in protein secretion systems type I, II, III, IV, and VI. Gene clusters involved in protein secretion were identified in a BLASTp search with protein sequences of gene clusters of secretion systems in P. aeruginosa. [file 12864_2015_1632_MOESM6_ESM.docx]

**Supplementary Table S1. Genes of WCS358, WCS374, and WCS417 putatively involved in protein secretion systems type I, II, III, IV, and VI.**

|  | Locus tags | Remark | Putative substrate |
| --- | --- | --- | --- |
| TISS | PS417_24400 - PS417_24390 |  | PS417-24405 |
|  | PS417_13460 - PS417_13470 |  | PS417_13450 (AprA)  PS417_13485 (lipase) |
|  | PD374_13210 - PD374_13220 |  | PD374_13200 (AprA)  PD374_13235 (lipase) |
|  | PD374_00665 - PD374_00675 |  | Unknown |
|  | PC358_01975 - PC358_01965 |  | Unknown |
|  | PC358_01710 - PC358_01705 |  | Unknown |
|  |  |  |  |
| T2SS | PS417_11255 - PS417_11305 | Hxc | PS417_11310 (alkaline phosphatase) |
|  | PS417_18035 - PS417_18085 | Xcp | PS417_18010 (acid phosphatase) |
|  | PD374_17220 - PD374_17270 | Xcp | PD374_17215 (acid phosphatase) |
|  | PC358_15880 - PC358_15930 | Xcp | PC358_15935 (UxpA)  PC358_15935 (UxpB) |
|  | PC358_03700 - PC358_03735 | Xcm |  |
|  |  |  |  |
| T3SS | PD374_03550 - PD374_03650 |  | suppl. table 2 |
|  | PS417-03420 - PS417_03555 |  | suppl. table 2 |
|  |  |  |  |
| T4SS | Not found in WCS genomes | |  |
|  |  |  |  |
| T5aSS/  autotransporters | PD374_10300 |  | NA |
|  | PD374_13225 | PspA Peptidase-S8 domain; homolog of EprS of *P. aeruginosa* | NA |
|  | PD374_13230 | PspB; Peptidase-S8 domain. | NA |
|  | PD374_14880 | Pertactin-like PL-2 domain | NA |
|  | PD374_14960 | Pertactin-like PL-1 domain | NA |
|  | PD374_05505 | Peptidase-S8 domain | NA |
|  | PD374_25000 | Esterase EstA | NA |
|  | PD374_26250 | Zinc-peptidase domain of M28 family; Homolog of aminopeptidase PA0328 (AaaA) | NA |
|  | PD374_27535 |  | NA |
|  | PD374_27540 | Very small autotransporter; 433 AA; passenger domain: ~125 AA | NA |
|  | PS417_13480 | PspB Peptidase-S8 domain | NA |
|  | PS417_13475 | PspA Peptidase-S8 domain; homolog of EprS of *P. aeruginosa*. | NA |
|  | PS417_07050 | Pertactin-like PL-1 domain | NA |
|  | PS417_28205 | Very small autotransporter; 430 AA; passenger domain ~125 AA. | NA |
|  | PS417_00770 |  | NA |
|  | PC358_00115 | Very small autotransporter 358 AA; passenger domain ~80 AA. | NA |
|  | PC358_24605 | Esterase EstA |  |
|  |  |  |  |
| T5bSS/TPS | PS417_00760 | TpsA carries DUF637 domain | PS417_00765 |
|  | PS417_03125 |  |  |
|  | PS417_11345 |  | PS417_11315 |
|  | PD374_00810 | TpsA carries DUF637 and PT-Hint domain | PD374_00815 |
|  | PD374_03230 |  | Unknown |
|  | PD374_14110 |  | PD374_14105 |
|  | PD374_21825 |  | PD374_21830 |
|  | PC358_15870 |  | PC358_15875 |
|  | PC358_26165 |  | Unknown |
|  |  |  |  |
| T5dSS | PS417_19940 | Patatin-like protein PlpD |  |
|  | PD374_19460 | Patatin-like protein PlpD |  |
|  | PC358_11610 | Patatin-like protein PlpD |  |
| T6SS | PS417_27905 - PS417_27995 | Pseudomonas T6SS Cluster 3 (Barret *et al.*, 2011) |  |
|  | PS417_15255 - PS417_15330 | Pseudomonas T6SS Cluster 1.1 (Barret *et al.*, 2011) |  |
|  | PD374_27230 - PD374_27300 | Pseudomonas T6SS Cluster 3 (Barret *et al.*, 2011) |  |
|  | PD374_12705 - PD374_12185 | Pseudomonas T6SS Cluster 1.1 (Barret *et al.*, 2011) |  |
|  | PC358_02120 - PC358_02195 | Pseudomonas T6SS Cluster 1.1 (Barret *et al.*, 2011) |  |

Gene clusters involved in protein secretion were identified in a BLASTp search with protein sequences of gene clusters of secretion systems in P. aeruginosa.
