# Supplementary material for: Unearthing the genomes of plant-beneficial Pseudomonas model strains WCS358, WCS374 and WCS417
Source: BMC Genomics. 2015 Jul 22;16(1):539. doi: 10.1186/s12864-015-1632-z (PMC4509608; doi:10.1186/s12864-015-1632-z)
Supplement: Supplementary file 8 — List of putative type III secreted effectors. Putative type III secreted effector genes were identified by searching for conserved Hrp (Rsp) “box motifs” in the promoter regions of putative effectors and exploring the N-terminal protein sequence of candidate effectors for features typical of type III secreted effectors (i.e. abundance of Ser and polar residues, acidic residues in the first 12 positions, and an aliphatic amino acid in position 3 or 4) [114, 115]. [file 12864_2015_1632_MOESM8_ESM.docx]

| Locus tag | Gene name | % G+C | Hrp box | | | | N-terminal residues | | | |
| --- | --- | --- | --- | --- | --- | --- | --- | --- | --- | --- |
|  |  |  | **Presence** | **position** | **sequence** | **Protein size** | **Serines** | **Acidic** | **Polar** | **Aliphatic** |
| PS417_09765 |  | 54.3 | Yes | -94 | C**GGAACC**GTTGTTTCCTGTGCA**CCAC**TCA | 303 | 4 | 0 | 22 | yes |
| PS417_12720 |  | 55.2 | Yes | -76 | T**GGAACC**TTACGAAGTGCCAAG**CCAC**TTA | 2133 | 4 | 0 | 28 | yes |
| PS417_12770 |  | 56.8 | Yes | -52 | A**GGAACC**CCACTCCACAAGAGG**CCAC**ACA | 94 | 2 | 1 | 27 | no |
| PS417_13220 |  | 67.1 | Yes | -373 | T**GGAACC**CGCGCCTGCACGCC**CCAC**GGG | 239 | 4 | 0 | 21 | yes |
| PS417_20780 |  | 55.8 | Yes | -684 | C**GGAACT**GGTTGGCCGAGAAA**CCAC**GGG | 155 | 2 | 0 | 21 | yes |
| PS417_03420 | ropE | 59.9 | Yes | -34 | T**GGAACC**AAACAGGGCGGATGC**CCAC**TAG | 1432 | 3 | 1 | 25 | no |
| PS417_06035 |  | 55.4 | Yes | -60 | C**GGAACC**GGCCGGGTGCGCTCA**CCAC**AGA | 114 | 5 | 0 | 22 | yes |
| PS417_06040 |  | 51.8 | Yes | -32 | T**GGAACC**GCTTTGACTTAAATG**CCAC**ATA | 64 | 0 | 1 | 25 | yes |
| PS417_07390 |  | 58.7 | Yes | -67 | T**GGAACC**TTTTTTCAAGGGGCT**CCAC**GCA | 1169 | 4 | 0 | 19 | yes |
| PS417_26495 | ExoU | 60 | No |  |  | 643 | 2 | 0 | 25 | yes |
| PS417_23510 | HopJ | 60.8 | No |  |  | 112 | 2 | 2 | 23 | yes |
|  |  |  |  |  |  |  |  |  |  |  |
| PD374_10455 |  | 57.4 | Yes | -72 | A**GGAACT**GATTCCGACGCAAAA**CCAC**ACA | 339 | 9 | 1 | 27 | yes |
| PD374_10460 |  | 63.1 | Yes | -211 | A**GGAACG**AATTACGCCCATTTG**CCAC**AGA | 55 | 3 | 1 | 21 | yes |
| PD374_10465 |  | 59.4 | Yes | -97 | C**GGAACT**GCCGAGTTCCTCCAA**CCAC**ACA | 305 | 8 | 1 | 25 | yes |
| PD374_12660 |  | 56.8 | Yes | -223 | T**GGAACC**ACAGGGCGAGTGGCGA**CCAC**GCA | 97 | 2 | 2 | 25 | no |
| PD374_06830 |  | 57 | Yes | -67 | T**GGAACC**ATCTTCATTCGTACG**CCAC**AGA | 78 | 7 | 0 | 22 | yes |
| PD374_06915 |  | 51.3 | Yes | -84 | G**GGAACC**TTTTTTTCAGGCTTG**CCAC**AGA | 79 | 6 | 0 | 21 | yes |
| PD374_25750 | ExoU | 60.2 | No |  |  | 639 | 8 | 1 | 25 | yes |
| PD374_26065 |  | 63.4 | Yes | -380 | A**GGAACT**GGTGTCCGCGGCTT**CCAC**CAA | 496 | 8 | 1 | 23 | no |
| PD374_20980 |  | 55.2 | Yes | -44 | T**GGAACG**CCCAACGCCCCTTGCG**CCAC**GCA | 365 | 7 | 1 | 24 | yes |
| PD374_23045 | HopJ | 58.7 | No |  |  | 112 | 2 | 1 | 22 | yes |
| PD374_21415 |  | 62.2 | Yes | -924 | A**GGAACA**GTTCGCCGCCCAGGC**CCAC**GGA | 177 | 7 | 0 | 19 | no |
| PD374_17010 |  | 54.6 | Yes | -98 | A**GGAACT**TTTCCGACCGCCTGG**CCAC**GCA | 376 | 5 | 0 | 26 | no |
| PD374_23880 |  | 51.7 | Yes | -158 | T**GGAACT**TTACCTACAGCATCA**CCAC**TCA | 949 | 3 | 3 | 24 | no |
| PD374_10035 |  | 53.5 | Yes | -36 | A**GGAACG**GCCTCCACCCGTAACA**CCAC**ATA | 575 | 6 | 1 | 30 | no |
| PD374_10025 |  | 51.5 | Yes | -36 | C**GGAACG**GTTTCGTCAGGCCCG**CCAC**ATA | 499 | 7 | 0 | 23 | no |

**Supplementary Table S2. List of putative type III secreted effectors.** Putative type III secreted effector genes were identified by searching for conserved Hrp (Rsp) “box motifs” in the promoter regions of putative effectors and exploring the N-terminal protein sequence of candidate effectors for features typical of type III secreted effectors (i.e. abundance of Ser and polar residues, acidic residues in the first 12 positions, and an aliphatic amino acid in position 3 or 4)[114,115]
